# Reshaping the therapeutic landscape of IgA nephropathy: a Bayesian network meta-analysis on the comparative efficacy and safety of immunosuppressants and targeted agents

**DOI:** 10.1186/s12882-026-04996-w

**Published:** 2026-04-24

**Authors:** Rulong Chen, Jinxin Zhang, Jiating Chen, Tingfei Xie, Yunpeng Xu, Zhaoyong Hu, Jihong Chen

**Affiliations:** 1 Department of Nephrology, The People’s Hospital of Baoan Shenzhen, The Second Affiliated Hospital of Shenzhen University, Shenzhen Hospital of Guangdong Provincial People’s Hospital, The Affiliated Baoan Hospital of Southern Medical University, Shenzhen Baoan Clinical Medical School of Guangdong Medical University, Shenzhen, Guangdong 518000 PR China; 2 Guangdong Key Laboratory of Nanomedicine, CAS Key Laboratory of Biomedical Imaging Science and System, Shenzhen Engineering Laboratory of Nanomedicine and Nanoformulations, CAS Key Lab for Health Informatics, Shenzhen Institutes of Advanced Technology, CAS-HK Joint Lab of Biomaterials, Chinese Academy of Sciences, Shenzhen, 518055 China; 3 Nephrology Division, Baylor College of Medicine, 1 Baylor Plaza, Houston, TX 77030 USA

**Keywords:** IgA nephropathy, Immunosuppressive agents, Targeted-release budesonide, Complement factor B inhibitor, Anti-APRIL monoclonal antibody

## Abstract

**Background:**

Comparative evidence between conventional immunosuppressants and emerging targeted therapies for IgA nephropathy (IgAN) is limited by a lack of head-to-head trials. We conducted a Bayesian network meta-analysis to compare efficacy and safety signals across available randomized evidence.

**Methods:**

PubMed, the Cochrane Library, Web of Science, Scopus, and Embase were searched from inception to March 2025 for randomized controlled trials in adults with biopsy-proven IgAN comparing methylprednisolone, mycophenolate mofetil (MMF), tacrolimus, targeted-release budesonide (Nefecon), iptacopan, or sibeprenlimab versus placebo or supportive care. Outcomes included serious adverse events (SAEs), estimated glomerular filtration rate (eGFR) slope, proteinuria, and urinary protein-to-creatinine ratio (UPCR). Bayesian random-effects models generated risk ratios or mean differences with 95% credible intervals, and surface under the cumulative ranking (SUCRA) probabilities were used to describe comparative rankings. Prespecified subgroup analyses by baseline eGFR (< 60 vs. ≥ 60 mL/min/1.73 m²) were assessed using interaction tests.

**Results:**

Seventeen trials were included. For SAEs, most comparisons were inconclusive because of sparse data and wide uncertainty intervals, although iptacopan showed a numerically lower point estimate for serious adverse events within the network. Subgroup analyses did not suggest effect modification by baseline eGFR. For kidney function, Nefecon showed the most favorable point estimate for eGFR slope, ranking highest in SUCRA analyses; however, credible intervals crossed the null and these rankings should be interpreted with caution given the sparse evidence network. For proteinuria outcomes, methylprednisolone showed the largest reductions versus placebo when assessed by UPCR, while iptacopan and sibeprenlimab were also associated with reductions; many between-treatment contrasts remained imprecise.

**Conclusions:**

Among evaluated therapies, Nefecon showed the most favorable point estimate for eGFR slope; however, this finding was imprecise and should be interpreted as hypothesis-generating rather than evidence of superiority. Overall, comparative estimates remain uncertain because of sparse networks, heterogeneous regimens, and limited follow-up. SUCRA rankings should be considered exploratory rather than evidence of treatment superiority. These findings support individualized treatment decisions and highlight the need for larger, longer-term, directly comparative trials with hard renal outcomes.

**Supplementary Information:**

The online version contains supplementary material available at 10.1186/s12882-026-04996-w.

## Introduction

Immunoglobulin A nephropathy (IgAN), first described by Berger and Hinglais in 1968, is the most common primary glomerulonephritis worldwide and a major contributor to chronic kidney disease (CKD) and end-stage kidney disease (ESKD) [1, 2]. Its clinical course varies considerably, with East Asian populations often exhibiting more aggressive disease [3]. Despite decades of research, therapeutic options for IgAN have long been limited to optimized supportive care with renin–angiotensin system (RAS) inhibitors and, in high-risk patients, systemic corticosteroids. While glucocorticoids can reduce proteinuria and delay progression, their widespread use is constrained by significant toxicity [4, 5]. The TESTING trial, for example, demonstrated that high-dose methylprednisolone was associated with an excess of serious adverse events, underscoring the persistent efficacy–toxicity dilemma in IgAN management [6, 7].

Advances in understanding IgAN pathogenesis have catalyzed the development of novel therapies designed to intervene at distinct points in the disease cascade, as conceptualized by the “four-hit hypothesis.” This model links mucosal overproduction of galactose-deficient IgA1, the generation of anti-glycan autoantibodies, formation of nephritogenic immune complexes, and subsequent complement activation. These mechanistic insights have yielded multiple therapeutic targets [8, 9]. Targeted-release budesonide (Nefecon) aims to suppress mucosal IgA1 production, iptacopan inhibits complement factor B to block the alternative pathway, and sibeprenlimab, a monoclonal antibody against APRIL, disrupts B-cell survival and IgA class switching [10, 11]. Together, these agents represent a new generation of mechanism-based therapies intended to achieve efficacy while limiting systemic toxicity.

At the same time, older immunosuppressive approaches—including methylprednisolone, mycophenolate mofetil (MMF), and tacrolimus—remain in clinical use despite variable efficacy and concerns regarding safety [12–14]. Comparative evidence between these established agents and newer targeted therapies remains scarce, as few head-to-head randomized controlled trials (RCTs) exist. This lack of direct evidence leaves clinicians without clear guidance for individualized treatment decisions.

Network meta-analysis (NMA) offers a rigorous method to address this gap by integrating both direct and indirect evidence across trials [15]. In this study, we conducted a systematic review and Bayesian NMA of 17 RCTs evaluating six therapeutic classes (including methylprednisolone, MMF, tacrolimus, Nefecon, iptacopan, and sibeprenlimab) in adult patients with IgAN. The analysis focused on outcomes most relevant to clinical decision-making: SAEs, estimated glomerular filtration rate (eGFR) slope, proteinuria, and urinary protein-to-creatinine ratio (UPCR). Subgroup analyses by baseline eGFR were further performed to explore heterogeneity in treatment response. By adopting a “traditional immunosuppressants versus novel targeted therapies” framework, this study aims to clarify the relative benefits and risks of available interventions and to inform more individualized treatment strategies for patients with IgAN. This network meta-analysis therefore focuses on comparing immunomodulatory agents—both conventional and novel against a common control of placebo or standard supportive care. This approach allows us to estimate the added benefit of immunosuppressive or targeted therapy in patients already receiving optimized background management.

## Methods

### Protocol and reporting guidelines

This network meta-analysis followed the Preferred Reporting Items for Systematic Reviews and Meta-Analyses extension for Network Meta-Analyses (PRISMA-NMA). The protocol was prospectively registered in PROSPERO (CRD420251087238), ensuring transparency and adherence to predefined methodology [16]. One amendment was made to the registered protocol after the commencement of the review.

### Search strategy

A comprehensive search was conducted in PubMed, the Cochrane Library, Web of Science, Scopus, and Embase from database inception through March 2025. Search terms combined disease-related keywords (“IgA nephropathy,” “Berger disease,” “glomerulonephritis”) with drug names and class descriptors of interest: systemic corticosteroids (e.g., methylprednisolone, prednisone), immunosuppressants (MMF, tacrolimus), targeted-release corticosteroids (Nefecon, TRF-budesonide), complement pathway inhibitors (iptacopan and other factor B inhibitors), and APRIL inhibitors (sibeprenlimab and related B-cell modulators). Reference lists of eligible RCTs and prior meta-analyses were also hand-searched.

### Study selection

Two reviewers independently screened titles and abstracts, followed by full-text review of potentially eligible studies. Disagreements were resolved by consensus or consultation with a third reviewer. Eligible trials were required to be randomized controlled trials enrolling adults (≥ 18 years) with biopsy-proven IgA nephropathy. All trials were required to be conducted on a background of optimized supportive care. Interventions included at least one of the six predefined therapies—methylprednisolone, MMF, tacrolimus, Nefecon, iptacopan, or sibeprenlimab—compared with placebo or supportive care. Studies had to report at least one prespecified outcome: SAEs, estimated glomerular filtration rate (eGFR) slope, proteinuria, or UPCR. Trials with mixed glomerulopathies were excluded unless IgAN-specific data could be extracted.Eligible trials were required to compare one of the six predefined immunomodulatory agents (methylprednisolone, MMF, tacrolimus, Nefecon, iptacopan, or sibeprenlimab) against placebo or standard supportive care.

### Data extraction and quality assessment

Data were extracted independently by two reviewers using a standardized template. Collected information included study characteristics (first author, year, country/region, sample size), baseline patient demographics and renal function, intervention details (drug, dosage, duration), and quantitative outcome data (means, standard deviations, sample sizes, or event counts). Risk of bias was assessed for each trial using the Cochrane Risk of Bias 2 (RoB 2) tool across five domains: randomization, deviations from intended interventions, missing outcome data, outcome measurement, and selective reporting. Disagreements were resolved through consensus [17]. When trials reported multiple dose arms of the same molecule, each dose arm was extracted separately under a single study identifier. For descriptive displays (forest plots) we also derived a within-trial, molecule-level combined arm as described below to avoid double-counting shared comparators. For eGFR slope, we extracted data as reported in each original study without re-calculation, accepting variability in definitions such as two-point versus multi-point calculation and differences in follow-up duration as inherent to the evidence base.

### Statistical analysis

We conducted an arm-based random-effects network meta-analysis in R. Continuous outcomes were modeled using a normal likelihood with identity link at the arm level and summarized as mean differences with 95% credible intervals. Dichotomous outcomes were modeled using a binomial likelihood at the arm level and summarized as risk ratios with 95% CrIs. Multi-arm trials were entered under a single study identifier and the correlation induced by shared comparators was accounted for in the model. Intervention nodes for the primary NMA were defined at the dose level [18]. Comparative rankings of interventions were derived using the surface under the cumulative ranking (SUCRA) probabilities. Between-study heterogeneity in the network meta-analysis was assessed using the between-study standard deviation (τ) derived from the posterior distributions of the Bayesian random-effects models. For each outcome, the median τ and its 95% credible interval are reported to characterize the degree of heterogeneity. For the conventional pairwise meta-analyses presented alongside the network results, heterogeneity was quantified using the I² statistic. Model convergence was confirmed by inspection of Markov chain Monte Carlo (MCMC) trace plots and Gelman–Rubin shrink factors, with diagnostic results presented in Supplementary Figures S2–S5. Consistency between direct and indirect evidence was assessed using node-splitting models when applicable. We conducted a sensitivity analysis by excluding studies with a high risk of bias, the results were consistent with the primary analysis. The potential for publication bias was assessed using comparison-adjusted funnel plots for each outcome. We planned to assess the overall certainty of evidence for each outcome using the GRADE approach for network meta-analysis.

To avoid double-counting and improve readability in study-level forest plots, multiple dose arms belonging to the same molecule within a trial were combined into a single treatment arm for that descriptive display only. For dichotomous outcomes, responders and sample sizes were summed within trial. For continuous outcomes, arms were pooled within trial using inverse-variance methods. The network meta-analysis and comparison‑adjusted funnel plots, however, were conducted at the dose-node level and therefore display separate points for different doses. Accordingly, the forest plots are descriptive, whereas NMA estimates, SUCRA, and small-study effect assessments reflect dose-specific nodes.

### Subgroup analyses

To explore potential effect modification, a prespecified subgroup analysis was conducted according to baseline kidney function, using an eGFR threshold of 60 mL/min/1.73 m². This threshold was chosen based on its clinical relevance as a marker of higher progression risk in IgAN and because it allowed for a balanced distribution of available data across subgroups within our network for meaningful comparison. Interaction P-values were used to test for subgroup differences, with *P* < 0.05 considered evidence of heterogeneity.

## Results

### Study selection

**Fig. 1:**
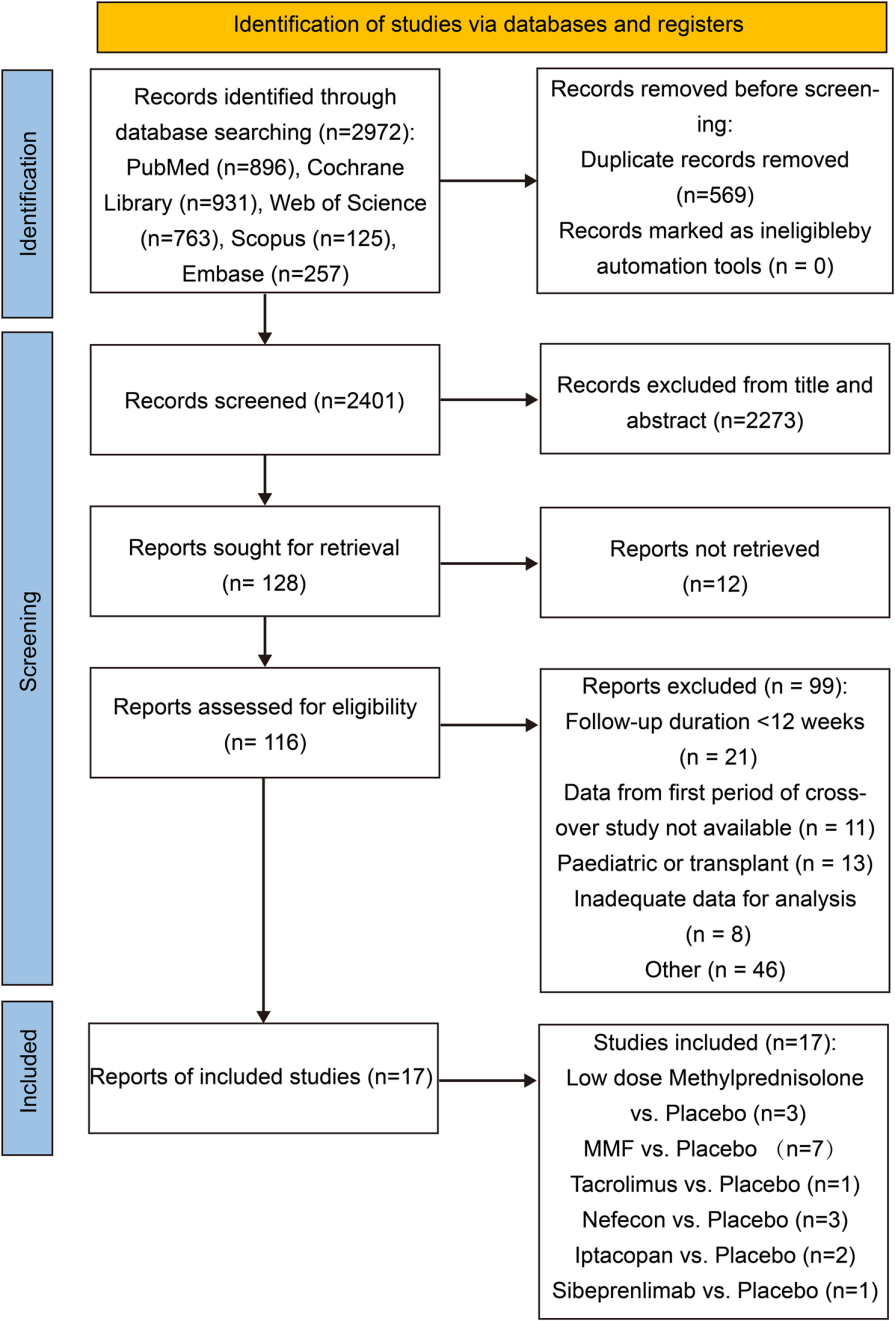
PRISMA flow diagram of study selection. A total of 17 randomized controlled trials (RCTs) in adult patients with IgA nephropathy, comparing methylprednisolone, mycophenolate mofetil (MMF), tacrolimus, targeted-release budesonide (Nefecon), iptacopan, or sibeprenlimab versus placebo or supportive care, were included after screening 2,972 records and applying predefined eligibility criteria

Our systematic search identified 2,972 records from PubMed (*n* = 896), Cochrane Library (*n* = 931), Web of Science (*n* = 763), Scopus (*n* = 125), and Embase (*n* = 257). After removal of 569 duplicates, 2,401 records were screened by title and abstract, of which 2,273 were excluded. Among the 128 full-text reports retrieved, 12 could not be accessed, leaving 116 for eligibility assessment. A further 99 were excluded, most commonly due to short follow-up duration (< 12 weeks, *n* = 21), lack of crossover first-period data (*n* = 11), pediatric or transplant populations (*n* = 13), insufficient data (*n* = 8), or other reasons (*n* = 46). The main other reasons included: non-randomized design (*n* = 17), duplicate publication (*n* = 11), trial protocol or interim analysis without outcome data (*n* = 12), and insufficient baseline or outcome data despite contacting authors (*n* = 6). Ultimately, 17 randomized controlled trials met the inclusion criteria (Fig. 1). These comprised studies evaluating low-dose methylprednisolone (*n* = 3), mycophenolate mofetil (MMF, *n* = 7), tacrolimus (*n* = 1), nefecon (*n* = 3), iptacopan (*n* = 2), and sibeprenlimab (*n* = 1), each compared with placebo [6, 19–34].

### Baseline characteristics of included trials

The 17 eligible trials enrolled patients with biopsy-proven primary IgA nephropathy and persistent proteinuria despite optimized supportive therapy. Median follow-up ranged from 6 to 72 months (mean ± SD: 24.5 ± 18.2 months), with most studies excluding secondary IgA nephropathy, systemic autoimmune disease, or rapidly progressive forms (Table S1). The distribution of follow-up durations across trials is illustrated in Supplementary Figure S6. Across studies, baseline demographics were broadly consistent (Table S2). Patients were typically young to middle-aged adults (mean age 30–45 years), with a male predominance of 50–70%. Body mass index was generally within the normal range (22–27 kg/m²). Renal function at enrollment reflected early to moderately impaired reserve, with mean eGFR values between 35 and 70 mL/min/1.73 m². Proteinuria or urine protein-to-creatinine ratio consistently exceeded 1 g/day or 1 g/g, indicating high-risk disease activity despite renin–angiotensin system inhibition. Notably, some cohorts included patients with relatively preserved kidney function (eGFR > 80 mL/min/1.73 m²), particularly in methylprednisolone and nefecon studies, underscoring efforts to intervene prior to irreversible decline. Together, these trials represent a heterogeneous but clinically meaningful spectrum of IgA nephropathy populations, balancing geographic and therapeutic diversity while maintaining consistent criteria for high-risk disease defined by sustained proteinuria.

### Risk of bias assessment

**Fig. 2:**
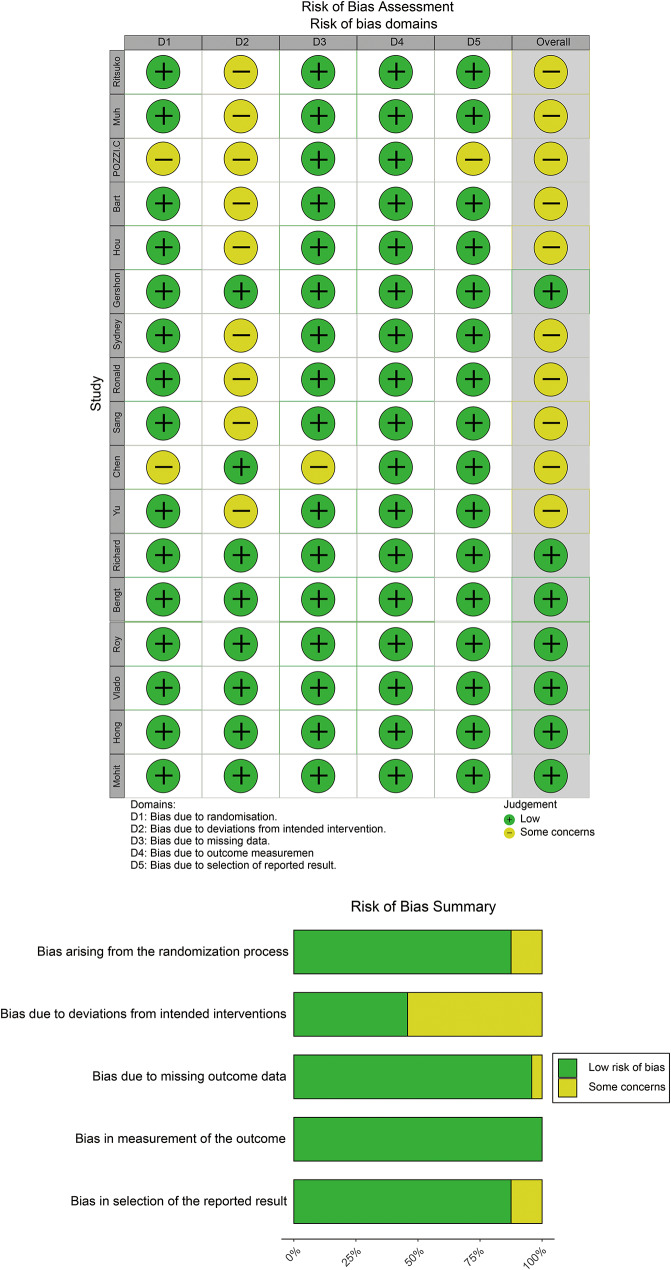
Risk of bias assessment using the Cochrane RoB 2 tool. Domains include randomization, deviations from intended interventions, missing outcome data, outcome measurement, and selective reporting

The methodological quality of the 17 included randomized controlled trials was appraised using the Cochrane Risk of Bias 2.0 tool (Fig. 2). Overall, randomization procedures (D1) were generally adequate, with only a small number of studies presenting some concerns due to limited reporting of sequence generation or allocation concealment. Deviations from intended interventions (D2) represented the main source of concern, with a substantial proportion of trials rated as some concerns, reflecting imperfect blinding and/or potential deviations from protocol. Missing outcome data (D3) and outcome measurement (D4) were uniformly low risk across all studies. Bias in the selection of reported results (D5) was predominantly low risk, with only a few studies raising some concerns. Taken together, the risk of bias across trials is best characterized as some concerns overall, driven mainly by D2, with other domains largely at low risk and no high‑risk judgments observed. These findings support the robustness of the evidence base while underscoring the importance of transparent reporting in future IgA nephropathy trials. Sensitivity analyses excluding studies with some concerns of bias yielded results consistent with the primary findings, supporting their robustness. Visual inspection of the comparison-adjusted funnel plots did not reveal substantial asymmetry, suggesting a low likelihood of major publication bias (Figure S1). The GRADE assessment method constitutes a comprehensive evaluation of the quality of evidence for each indicator across all treatment groups (Table S3).

### Incidence of serious adverse events

**Fig. 3:**
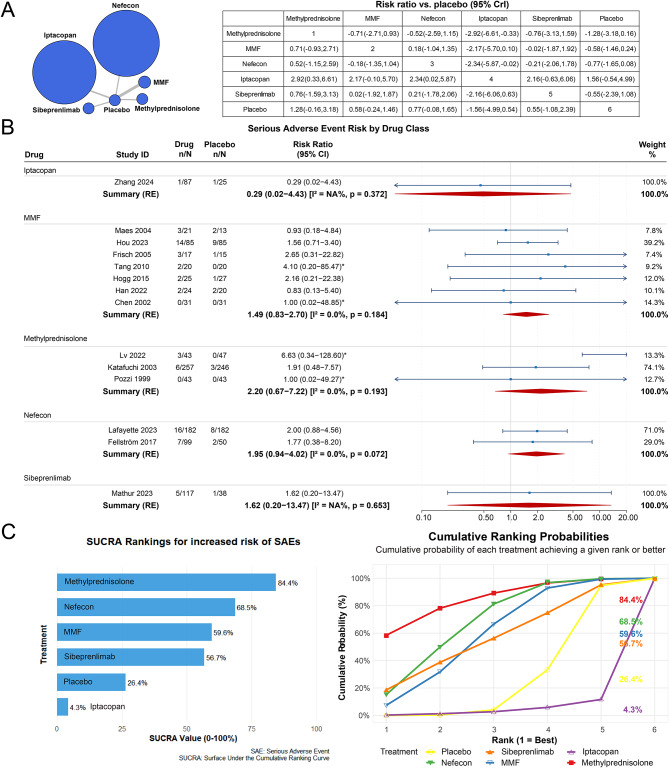
Network meta-analysis of serious adverse events (SAEs). (**A**) Network geometry of available treatment comparisons. The size of each node is proportional to the total patient sample size allocated to that treatment group. (**B**) Forest plots of risk ratios versus placebo. Rist ratios with 95% credible intervals (CIs) are shown from pairwise random-effects meta-analyses. (**C**) Surface under the cumulative ranking curve (SUCRA) ranking probabilities of interventions for safety

The network meta-analysis compared the risk of serious adverse events (SAEs) across methylprednisolone, Nefecon, mycophenolate mofetil (MMF), iptacopan, sibeprenlimab, and placebo. Overall, most between-treatment comparisons were inconclusive, with 95% credible intervals (CrIs) overlapping the null, reflecting sparse event data and substantial uncertainty. In the network estimates, iptacopan showed a numerically lower point estimate for SAE risk compared with other interventions, representing a favorable safety signal rather than definitive evidence of reduced risk (Fig. 3A). In conventional pairwise random-effects meta-analyses versus placebo (frequentist framework), the pooled estimates for SAEs were: iptacopan (RR 0.29, 95% CI 0.02–4.43; I² = NA), MMF (RR 1.49, 95% CI 0.83–2.70; I² = 0%), methylprednisolone (RR 2.20, 95% CI 0.67–7.22; I² = 0%), Nefecon (RR 1.95, 95% CI 0.94–4.02; I² = 0%), and sibeprenlimab (RR 1.62, 95% CI 0.20–13.47; I² = NA) (Fig. 3B). Although the point estimate for iptacopan versus placebo suggested a lower risk of SAEs, the confidence interval was extremely wide, indicating profound uncertainty and precluding any definitive conclusions regarding comparative safety. Based on SUCRA values for SAE risk, methylprednisolone ranked highest for increased risk (84.4%), followed by Nefecon (68.5%), MMF (59.6%), and sibeprenlimab (56.7%), whereas placebo ranked lower (26.4%). Iptacopan ranked lowest (4.3%), corresponding to the most favorable position in the probabilistic SUCRA ranking (Fig. 3C). However, given that several nodes are supported by only 1–2 trials, these rankings are highly sensitive to sparse data and should be interpreted as exploratory rather than evidence of a true safety hierarchy. The wide uncertainty intervals and limited number of SAE events further preclude definitive conclusions. Convergence of the Bayesian safety model was supported by inspection of trace plots and Gelman–Rubin shrink factors approaching 1.0, with stable trace lines and unimodal posterior distributions shown in Supplementary Figure S2. The estimated between-study standard deviation τ for SAEs was 0.20 (95% CrI: 0.01 to 0.77), indicating low heterogeneity across trials contributing to this network. Prespecified subgroup analyses by baseline eGFR (< 60 vs. ≥ 60 mL/min/1.73 m²) did not suggest meaningful effect modification on SAE risk (Table S4). For MMF, SAE risk estimates were similar across eGFR strata (RR 1.77, 95% CI 0.45–6.98 for eGFR < 50; RR 1.33, 95% CI 0.75–2.35 for eGFR ≥ 50; P for interaction = 0.62). For other interventions, subgroup-specific estimates were unavailable for at least one stratum because of sparse data, limiting formal comparisons. Overall, these findings suggest that baseline kidney function did not materially modify SAE risk within the available evidence base. Qualitative assessment of adverse event profiles showed that methylprednisolone was associated with a higher frequency of steroid-related events, including impaired glucose tolerance or new-onset diabetes, insomnia, and infections such as pneumonia. MMF was more commonly associated with gastrointestinal adverse effects (e.g., diarrhea and nausea) and leukopenia. For newer targeted therapies, preliminary safety data for iptacopan did not reveal prominent drug-specific adverse event patterns within the limited follow-up available, whereas safety data for sibeprenlimab remained limited. These observations are consistent with known pharmacologic mechanisms and provide contextual background for interpreting the overall risk–benefit profiles of the evaluated therapies.

### Comparative efficacy of interventions in preserving kidney function

**Fig. 4:**
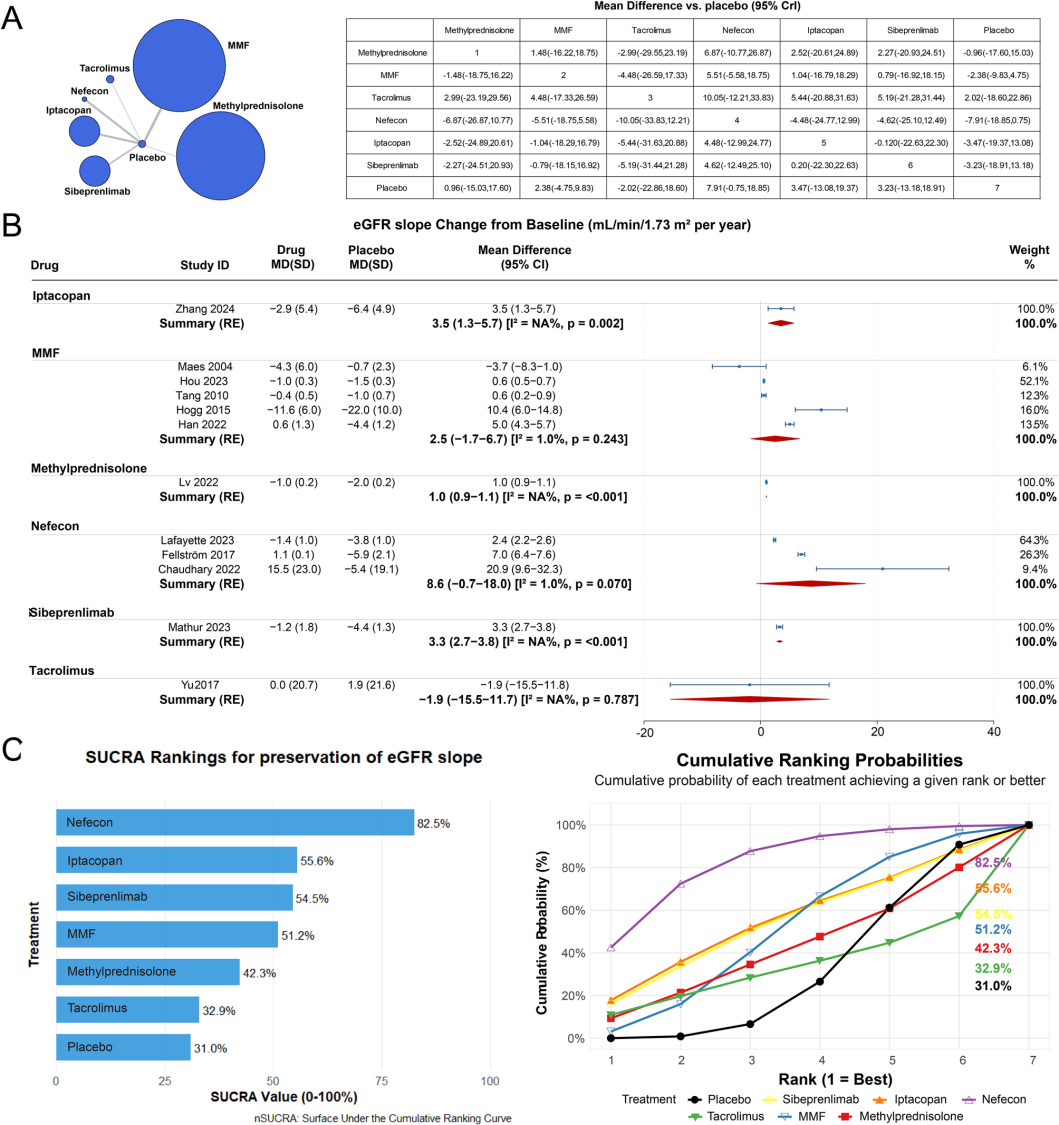
Network meta-analysis of estimated glomerular filtration rate (eGFR) slope. (**A**) Network geometry of treatment comparisons. The size of each node is proportional to the total patient sample size allocated to that treatment group. (**B**) Forest plots of mean differences in eGFR slope versus placebo. Mean differences with 95% credible intervals (CIs) are shown from pairwise random-effects meta-analyses. (**C**) Surface under the cumulative ranking curve (SUCRA) rankings of interventions for kidney function preservation

A network meta-analysis was performed to compare changes in eGFR slope from baseline across methylprednisolone, MMF, tacrolimus, Nefecon, iptacopan, sibeprenlimab, and placebo. Based on the NMA estimates, Nefecon was associated with the greatest improvement in eGFR slope change compared with placebo (mean difference [MD] 7.91, 95% CrI − 0.75–18.85). Sibeprenlimab (MD 3.23, 95% CrI − 13.18–18.91) and iptacopan (MD 3.47, 95% CrI − 13.08–19.37) showed numerically positive point estimates relative to placebo; however, the 95% credible intervals for all therapies crossed 0, indicating no statistically credible benefit (Fig. 4A). In conventional pairwise random-effects meta-analyses versus placebo, the pooled effects on eGFR slope change from baseline were: iptacopan (MD 3.5, 95% CI 1.3–5.7; I² = NA), MMF (MD 2.5, 95% CI − 1.7 to 6.7; I² = 1.0%), methylprednisolone (MD 1.0, 95% CI 0.9–1.1; I² = NA), Nefecon (MD 8.6, 95% CI -0.7–18.0; I² = 1.0%), sibeprenlimab (MD 3.3, 95% CI 2.7–3.8; I² = NA), and tacrolimus (MD − 1.9, 95% CI − 15.5 to 11.7; I² = NA) (Fig. 4B). Ranking for preservation of eGFR slope decline placed Nefecon first (82.5%), followed by iptacopan (55.6%), sibeprenlimab (54.5%), MMF (51.2%) and methylprednisolone (42.3%). Tacrolimus (32.9%) and placebo (31.0%) had the lowest rankings (Fig. 4C). However, these probabilistic rankings are based on a sparse network with limited trials per node and should be viewed as hypothesis-generating rather than evidence of differential efficacy. Model convergence for the Bayesian analysis was confirmed by trace plots and Gelman–Rubin shrink factors approaching 1.0. Supplementary Figure S3 demonstrated stable trace lines and unimodal posterior distributions, supporting the reliability of the eGFR slope estimates. For the eGFR slope network, the estimated τ was 6.56 (95% CrI: 3.16 to 15.44) mL/min/1.73 m² per year, reflecting the expected variability in follow-up durations and study populations across the included trials. Subgroup analyses stratified by baseline eGFR (< 60 vs. ≥ 60 mL/min/1.73 m²) were performed to assess whether baseline renal function modified treatment effects on eGFR slope change (Table S4). The direction and magnitude of effects were generally consistent with the overall network estimates, and no statistically significant subgroup differences were detected based on interaction testing. However, several treatment–subgroup cells were informed by limited data, resulting in wide intervals and reducing power to detect effect modification. Therefore, the subgroup findings should be interpreted as exploratory and primarily supportive of the main results.

### Comparative effects of interventions on proteinuria change

**Fig. 5:**
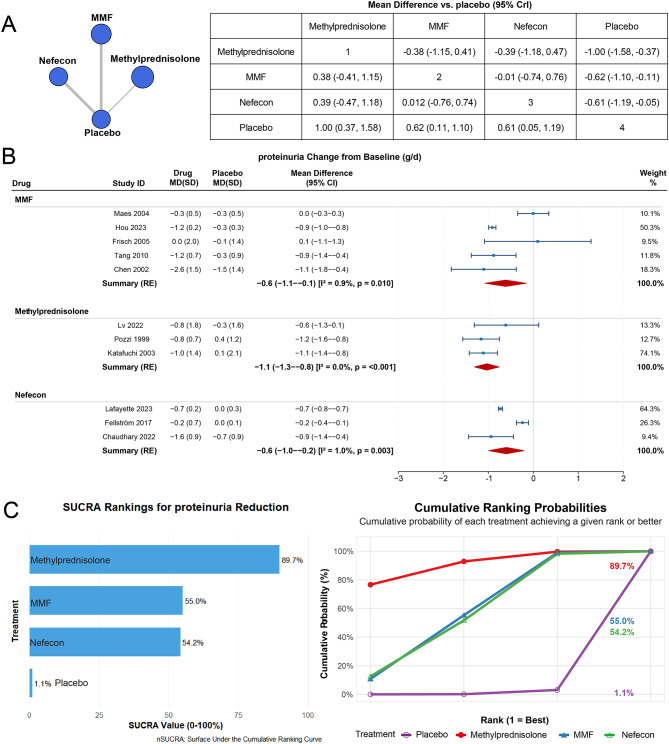
Network meta-analysis of proteinuria change. (**A**) Network geometry of treatment comparisons. (**B**) Forest plots of mean differences in proteinuria versus placebo. The size of each node is proportional to the total patient sample size allocated to that treatment group. Mean differences with 95% credible intervals (CIs) are shown from pairwise random-effects meta-analyses. (**C**) Surface under the cumulative ranking curve (SUCRA) rankings of interventions for proteinuria reduction

Separate network meta-analyses were conducted for 24-hour proteinuria and UPCR, as these outcomes represent distinct measures of proteinuria and are not interchangeable. A network meta-analysis compared the change in proteinuria from baseline (g/day) across methylprednisolone, mycophenolate mofetil, Nefecon, and placebo. In the NMA, methylprednisolone was associated with the greatest reduction and was superior to placebo (methylprednisolone vs. placebo: MD − 1.00, 95% CrI − 1.58 to − 0.37). MMF and Nefecon tended to be less effective than methylprednisolone, although the differences were not statistically significant (MMF vs. methylprednisolone: MD 0.38, 95% CrI − 0.41 to 1.15; Nefecon vs. methylprednisolone: MD 0.39, 95% CrI − 0.47 to 1.18) (Fig. 5A). In pairwise random-effects meta-analyses versus placebo, the pooled mean differences were: methylprednisolone (MD − 1.1, 95% CI − 1.3 to − 0.8; I² = 0.0%), MMF (MD − 0.6, 95% CI − 1.1 to − 0.1; I² = 0.9%), and Nefecon (MD − 0.6, 95% CI − 1.0 to − 0.2; I² = 1.0%) (Fig. 5B). Based on SUCRA values for proteinuria reduction, methylprednisolone ranked first (89.7%), followed by MMF (55.0%), Nefecon (54.2%), and placebo (1.1%) (Fig. 5C). The cumulative ranking curves were consistent with these rankings. However, given the limited number of trials informing several nodes, particularly for MMF and Nefecon in this proteinuria network, these SUCRA rankings should be interpreted with caution and considered exploratory rather than evidence of differential efficacy. Model convergence for the Bayesian proteinuria analysis was confirmed by trace plots and Gelman–Rubin shrink factors, with Supplementary Figure S4 demonstrating stable trace lines and unimodal posterior distributions. The between-study standard deviation τ for the proteinuria network was 0.40 (95% CrI: 0.21 to 0.85) g/day, suggesting moderate heterogeneity.

**Fig. 6:**
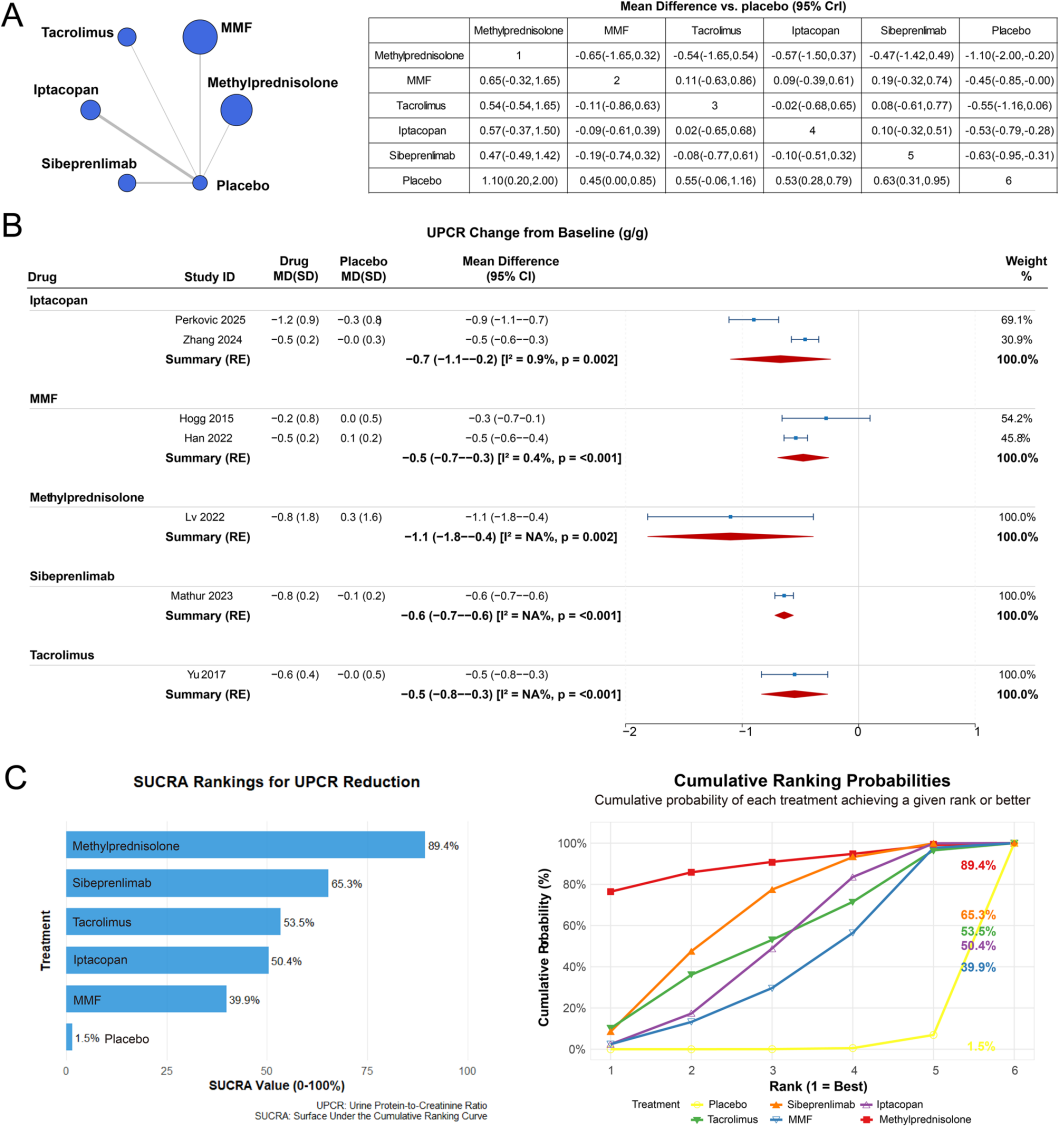
Network meta-analysis of urinary protein-to-creatinine ratio (UPCR). (**A**) Network geometry of treatment comparisons. The size of each node is proportional to the total patient sample size allocated to that treatment group. (**B**) Forest plots of mean differences in UPCR versus placebo. Mean differences with 95% credible intervals (CIs) are shown from pairwise random-effects meta-analyses. (**C**) Surface under the cumulative ranking curve (SUCRA) rankings of interventions for UPCR reduction

A network meta-analysis (NMA) was conducted to compare the change in urine protein-to-creatinine ratio (UPCR) from baseline across methylprednisolone, mycophenolate mofetil, tacrolimus, iptacopan, sibeprenlimab, and placebo. In the NMA league table, methylprednisolone showed greater UPCR reduction than placebo (MD − 1.10, 95% CrI − 2.00 to − 0.20). Compared with placebo, UPCR was also reduced with MMF (MD − 0.45, 95% CrI − 0.84 to 0.03), iptacopan (MD − 0.53, 95% CrI − 0.79 to − 0.28), and sibeprenlimab (MD − 0.63, 95% CrI − 0.95 to − 0.31), whereas tacrolimus did not show a clear difference (MD − 0.55, 95% CrI − 1.16 to 0.06). Between active treatments, most contrasts were imprecise, although methylprednisolone tended to yield larger UPCR reductions than other interventions (Fig. 6A). In conventional pairwise random-effects meta-analyses versus placebo, the pooled effects on UPCR change from baseline (g/g) were: iptacopan (MD − 0.7, 95% CI − 1.1 to − 0.2; I² = 0.9%), MMF (MD − 0.5, 95% CI − 0.7 to − 0.3; I² = 0.4%), methylprednisolone (MD − 1.1, 95% CI − 1.8 to − 0.4; I² = NA), sibeprenlimab (MD − 0.6, 95% CI − 0.7 to − 0.6; I² = NA), and tacrolimus (MD − 0.5, 95% CI − 0.8 to − 0.3; I² = NA) (Fig. 6B). Ranking based on SUCRA values for UPCR reduction placed methylprednisolone first (SUCRA 89.4%), followed by sibeprenlimab (65.3%), tacrolimus (53.5%), iptacopan (50.4%), and MMF (39.9%), while placebo ranked last (1.5%) (Fig. 6C). The cumulative ranking curves were consistent with these findings. It should be emphasized, however, that several treatments in this UPCR network are supported by only one or two trials; therefore, these probabilistic rankings are inherently unstable and should be viewed as hypothesis-generating rather than evidence of comparative superiority. Model convergence for the Bayesian UPCR analysis was confirmed by trace plots and Gelman–Rubin shrink factors (Figure S5). For the UPCR network, τ was estimated at 0.22 (95% CrI: 0.12 to 0.46) g/g, indicating low to moderate heterogeneity.

We noted a difference in the magnitude of proteinuria reduction attributed to MMF between studies using 24-hour collection and those using UPCR. Studies reporting 24-hour proteinuria generally enrolled patients with higher baseline proteinuria and more impaired renal function, which may explain their larger observed treatment effects. Additionally, the greater biological variability inherent in spot urine UPCR measurements compared to 24-hour collections could lead to attenuation of the estimated effect size. Therefore, each set of results is best interpreted within the context of its respective trial population and methodology.

## Discussion

### Summary of evidence

This Bayesian network meta-analysis of randomized trials in adults with IgA nephropathy compared the efficacy and safety of methylprednisolone, conventional immunosuppressants, and newer mechanistic therapies using serious adverse events and commonly reported renal surrogate outcomes. Within the network, Nefecon yielded the most favorable point estimate for eGFR slope, although the associated uncertainty remained substantial and the credible interval crossed the null. This signal was driven largely by the pivotal NefIgArd trial, which reported improvement in total eGFR slope over 24 months. However, this finding should be interpreted cautiously, because total eGFR slope was not uniformly defined across trials and may reflect early treatment-related effects in addition to sustained disease-modifying benefit. For proteinuria assessed by 24-hour urinary protein excretion (g/day), methylprednisolone showed the largest reduction versus placebo. MMF and Nefecon were also associated with reductions in proteinuria, although the magnitude of effect appeared smaller than that observed with methylprednisolone. For UPCR, methylprednisolone again showed a favorable reduction versus placebo, and iptacopan and sibeprenlimab also showed potentially beneficial signals. These proteinuria outcomes were analyzed separately because they reflect different measurement methods and are not directly interchangeable across trials. Across efficacy outcomes, many comparisons remained imprecise, with several credible intervals crossing the null. Accordingly, these findings should be interpreted as comparative signals within a sparse evidence network rather than as definitive treatment rankings. These results suggest that different therapies may show relative advantages across different surrogate outcomes, while no regimen can yet be regarded as clearly superior across all domains. Treatment selection in IgA nephropathy therefore still requires balancing potential kidney benefit against regimen-specific toxicity, feasibility, and cost. This is consistent with the current approach to IgA nephropathy management, in which optimized supportive care remains the foundation of therapy and immunomodulatory treatment is generally reserved for patients at higher risk of progression [35, 36].

### Clinical implications

From a clinical perspective, these findings highlight familiar therapeutic trade-offs. For kidney function, Nefecon showed the most favorable point estimate within this sparse network, but this signal was imprecise and should not be interpreted as definitive evidence of superiority over other regimens. For proteinuria reduction, methylprednisolone showed the largest effect in analyses using 24-hour urinary protein excretion, whereas MMF and Nefecon also demonstrated favorable signals. In analyses based on UPCR, methylprednisolone again appeared favorable, with iptacopan and sibeprenlimab also showing potentially beneficial effects. These differences should be interpreted cautiously, because the outcomes were measured differently across trials and the evidence base remained limited for several treatment nodes. For iptacopan, point estimates suggested a potentially favorable safety profile, but the available evidence was too limited to conclude that it is safer than placebo or other active therapies. This distinction is important, because overinterpretation of imprecise safety signals may lead to misleading clinical inferences [37].

Cost and access will also influence real-world implementation. Newer targeted agents may be substantially more expensive than conventional options, and affordability and reimbursement vary widely across health care settings. Even when efficacy signals appear promising, uptake may depend on cost-effectiveness and budget impact, particularly because IgA nephropathy is a chronic disease that often requires prolonged treatment and monitoring [38, 39].

The comparative safety estimates from this network meta-analysis, particularly for serious adverse events, were associated with considerable uncertainty because of low event rates and sparse data for several interventions. Accordingly, SUCRA rankings and point estimates should not be interpreted as evidence of a true safety hierarchy. At most, they provide hypothesis-generating signals that require confirmation in larger and longer-term studies. In current practice, treatment-related safety should still be judged primarily on direct evidence from pivotal trials.

### Strengths and limitations

This study followed a registered protocol, used a comprehensive search strategy, applied a standardized risk-of-bias assessment, and implemented Bayesian random-effects network models with convergence diagnostics and consistency assessment. We evaluated both safety and multiple renal surrogate outcomes commonly used in IgAN trials [40, 41].

The main limitations arise from the underlying evidence base. Several interventions were informed by only one or two trials, resulting in wide credible intervals and making SUCRA rankings sensitive to sparse data. Interpretation of eGFR slope was particularly challenging across the included trials. Definitions of slope were not uniform, with differences in calculation methods, follow-up duration, and endpoint reporting. Most studies reported total eGFR slope without separating acute and chronic phases. This distinction is important because corticosteroid-based therapies may induce early hemodynamic or anti-inflammatory effects that transiently increase eGFR after treatment initiation, thereby influencing total slope estimates and potentially overstating long-term disease-modifying benefit. This issue is especially relevant when interpreting the favorable point estimate observed for Nefecon in our network, because that estimate was derived from trial-reported total slope over relatively limited follow-up and without consistent phase separation. Accordingly, differences in total eGFR slope across treatments should be interpreted cautiously and should not be considered definitive evidence of sustained kidney protection.

Our findings should be interpreted alongside the recent analyses by Kim et al., including both the class-based meta-analysis in CJASN and the post hoc TESTING analysis in Kidney International Reports [46, 47]. These studies reinforce the importance of corticosteroid-based therapy in IgA nephropathy and indicate that reduced-dose methylprednisolone in TESTING was associated with meaningful improvement in proteinuria and total eGFR slope, albeit with important safety trade-offs. In contrast, our Bayesian network meta-analysis was designed to compare regimen-level nodes across a sparse network and therefore addressed a different question from class-based pooling. The more favorable point estimate observed for Nefecon in our network should not be interpreted as evidence of superiority over moderate-dose methylprednisolone, because this estimate was imprecise, relied on limited direct evidence, and was influenced by heterogeneity in endpoint definition across trials. Interpretation of total eGFR slope is further complicated by the fact that most studies did not separate acute and chronic slope phases; early hemodynamic or anti-inflammatory effects of glucocorticoid-based therapies may therefore influence total slope estimates. Accordingly, all comparative estimates for kidney function preservation should be regarded as exploratory rather than definitive, and direct long-term comparative trials remain necessary.

Additional limitations should also be acknowledged. Variation in methylprednisolone regimens could not be fully modeled, and regimen-level differences may influence both efficacy and safety [42–45]. Our study design was based on a shared background of optimized supportive care, allowing evaluation of the incremental effects of immunomodulatory therapies. However, supportive care continues to evolve, and specific protocols differed across trials. These differences likely reflect real-world practice, but they also mean that treatment effects should be interpreted within the supportive-care context of each study.

The safety analysis also has important limitations. The network was sparse and underpowered to detect true differences in rare events, and inconsistent reporting across trials prevented meaningful quantitative synthesis of several clinically relevant adverse outcomes beyond serious adverse events. Accordingly, comparative safety estimates, including SUCRA rankings, should be interpreted as exploratory rather than definitive. Our network included one study published only as a conference abstract [31]. Although it met our inclusion criteria and contributed geographic diversity, its preliminary nature should be acknowledged. Sensitivity analyses showed that exclusion of this study did not materially change the main conclusions. Most included trials reported efficacy only during the active treatment period and lacked systematic follow-up after treatment discontinuation. This limits assessment of the durability of treatment effects, particularly for short-course regimens in which early benefit may not necessarily reflect sustained disease modification. Post-treatment trajectories, including rebound proteinuria or subsequent changes in eGFR slope after withdrawal, could not be evaluated. Dose-level nodes were retained to preserve clinically meaningful differences in regimen intensity; however, this strategy may also further fragment an already sparse network and contribute to ranking instability. Finally, several emerging agents currently in early-phase development, including atacicept, telitacicept, zigakibart, and povedticept, were not included because mature phase III data are not yet available. Future network meta-analyses incorporating these data will be needed to provide a more complete therapeutic landscape.

### Conclusions and implications for future research

Future randomized trials should compare newer targeted therapies against contemporary standard care with clearly specified and comparable background therapy, including renin–angiotensin system blockade and other supportive measures that have become standard in recent years [36]. When systemic corticosteroids are evaluated, trials should report dose, route, tapering strategy, and treatment duration in sufficient detail and, where feasible, should directly compare regimen-specific approaches to better define the balance between efficacy and toxicity [5]. Longer follow-up, including post-treatment observation after treatment discontinuation, together with systematic reporting of hard kidney outcomes, is needed to determine whether short-term changes in proteinuria and total eGFR slope translate into durable clinical benefit [5, 44]. Future studies should also clarify optimal treatment duration, including whether short-course or prolonged therapy is required, particularly in light of the cost and access challenges associated with newer agents. Standardized endpoint definitions and more consistent adverse-event reporting would further strengthen future evidence synthesis, including more reliable network meta-analyses [40, 41]. Biomarker-guided stratification may help align patients with therapies targeting dominant pathogenic pathways, including complement activation and mucosal immune activity [48]. Pathology-based risk stratification frameworks, such as the Oxford classification, also remain important for trial design and interpretation, particularly when enriching for patients at higher risk of progressive disease [49].

Across the currently available randomized evidence, comparative estimates for both efficacy and safety remain subject to substantial uncertainty because of sparse networks, heterogeneous regimens, and limited and variable follow-up. Comparative inference for kidney function, as assessed by eGFR slope, was particularly challenging. Apparent differences in point estimates or probabilistic rankings should therefore be interpreted as exploratory rather than as evidence of definitive comparative superiority. This is especially important because most trials reported total eGFR slope without consistent separation of acute and chronic phases, and early treatment-related effects may influence slope estimates. Given the sparse network, with many treatment nodes informed by only one or two trials, these rankings are inherently unstable and should not be used in isolation to guide clinical decision-making. Although several therapies were associated with improvement in proteinuria-related outcomes, these surrogate effects varied in magnitude and precision across agents and did not consistently establish durable kidney benefit. Methylprednisolone showed the most consistent and statistically credible reductions in proteinuria, but its higher risk of serious adverse events underscores the central trade-off between surrogate efficacy and treatment tolerability.

Taken together, these findings argue against uniform treatment recommendations and instead support individualized decision-making based on disease severity, risk profile, safety considerations, treatment access, and patient preferences. Future research should prioritize larger, longer-term, directly comparative trials with standardized regimens, stage-stratified enrollment, and hard renal outcomes, together with rigorous evaluation of safety and cost-effectiveness, to more clearly define the long-term benefits and risks of both conventional immunosuppressants and emerging targeted therapies in IgA nephropathy.

## Electronic supplementary material

Below is the link to the electronic supplementary material.

Supplementary Material 1

## Data Availability

All extracted and calculated data are available by emailing to the corresponding author on reasonable request.

## Abbreviations

APRIL: A proliferation-inducing ligand
CAS: Chinese Academy of Sciences
CAS-HK: Chinese Academy of Sciences–Hong Kong
CI: Confidence interval
CKD: Chronic kidney disease
CrI: Credible interval
eGFR: Estimated glomerular filtration rate
ESKD: End-stage kidney disease
IgAN: Immunoglobulin A nephropathy
IgA: Immunoglobulin A
MCMC: Markov chain Monte Carlo
MD: Mean difference
MMF: Mycophenolate mofetil
NMA: Network meta-analysis
PRISMA-NMA: Preferred Reporting Items for Systematic Reviews and Meta-Analyses extension for Network Meta-Analyses
PROSPERO: International Prospective Register of Systematic Reviews
RAS: Renin-angiotensin system
RCT: Randomized controlled trial
RoB 2: Risk of Bias 2
RR: Risk ratio
SAE: Serious adverse event
SD: Standard deviation
SUCRA: Surface under the cumulative ranking curve
TRF: Targeted-release formulation
UPCR: Urinary protein-to-creatinine ratio
USA: United States of America
τ: Between-study standard deviation
