# Supplementary material for: Reshaping the therapeutic landscape of IgA nephropathy: a Bayesian network meta-analysis on the comparative efficacy and safety of immunosuppressants and targeted agents
Source: BMC Nephrol. 2026 Apr 24;27:359. doi: 10.1186/s12882-026-04996-w (PMC13267211; doi:10.1186/s12882-026-04996-w)
Supplement: Supplementary file 1 — Supplementary Material 1 [file 12882_2026_4996_MOESM1_ESM.docx]

**Reshaping the therapeutic landscape of IgA nephropathy: a Bayesian network meta-analysis on the comparative efficacy and safety of immunosuppressants and targeted agents**

Rulong Chen^1,2^, Jinxin Zhang^1,2^, Jiating Chen^1,2^, Tingfei Xie^1,2^, Yunpeng Xu^1^, Zhaoyong Hu^3^, Jihong Chen^1*^

^1^ Department of Nephrology, The People’s Hospital of Baoan Shenzhen, The Second Affiliated Hospital of Shenzhen University, Shenzhen Hospital of Guangdong Provincial People's Hospital, The Affiliated Baoan Hospital of Southern Medical University, Shenzhen Baoan Clinical Medical School of Guangdong Medical University, The 8th people’s Hospital of Shenzhen, Baoan Clinical Research Center for Kidney Disease, Guangdong 518000, PR China.

^2^ Guangdong Key Laboratory of Nanomedicine, CAS-HK Joint Lab of Biomaterials, CAS Key Laboratory of Biomedical Imaging Science and System, Shenzhen Engineering Laboratory of Nanomedicine and Nanoformulations, CAS Key Lab for Health Informatics, Shenzhen Institutes of Advanced Technology, Chinese Academy of Sciences, Shenzhen, 518055, China

^3^ Nephrology Division, Baylor College of Medicine, 1 Baylor Plaza, Huston, Texas, USA. 77030

Running Head: NMA of Therapies for IgA Nephropathy

*Corresponding authors:

Jihong Chen, E-mail: [chenjihong0606@hotmail.com](mailto:chenjihong0606@hotmail.com)

Table of Contents:

1. Figure S1….....…………………………..............…………………………………………S3

2. Figure S2…………………..………………..……....……..…………………........…….…S4

3. Figure S3……………………...................................…............………………...…….....S5

4. Figure S4…………………….................................................................……..…….… S6

5. Figure S5…………………….................................................................……..…….… S7

6. Table S1……………………...................................................................……..…….… S8

6. Table S2…………………….....................................................................…..…….… S13

7. Table S3…………………….................................................................……..…….… S15

8. Table S4…………………….................................................................……..…….… S16

**Supplementary Figures and Tables**


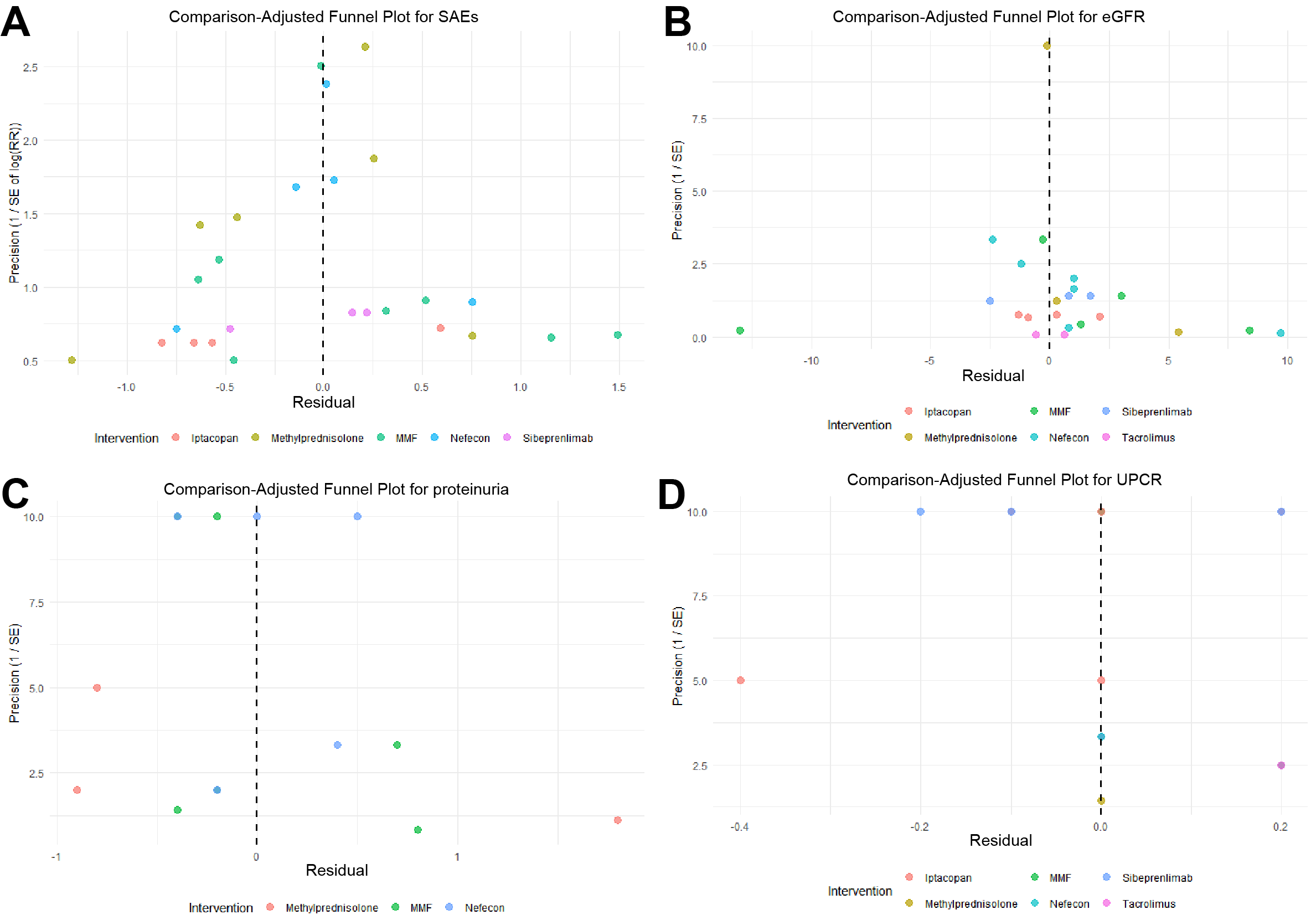


**Figure S1. Comparison-adjusted funnel plots assessing potential publication bias.**

Comparison-adjusted funnel plots for the outcomes of (A) serious adverse events (SAEs), (B) estimated glomerular filtration rate (eGFR), (C) proteinuria, and (D) urinary protein-to-creatinine ratio (UPCR).


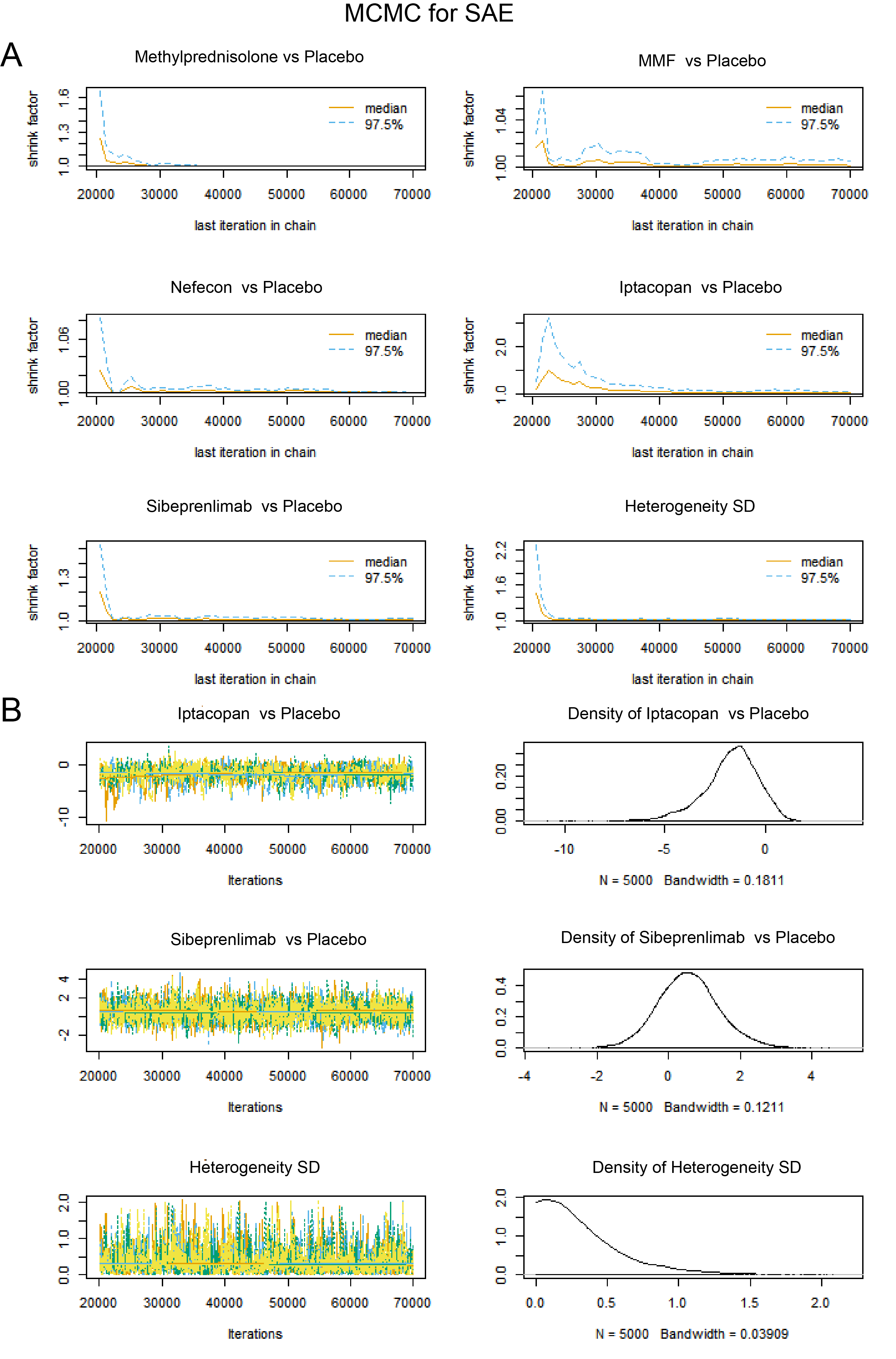


**Figure S2. Bayesian model diagnostics for the SAEs network.**

(A) Brooks–Gelman–Rubin diagnostic plot (Gelman plot) showing shrink factors close to 1.0; (B) Trace plots (left) and posterior density distributions (right) demonstrating satisfactory convergence of Markov chain Monte Carlo simulations.


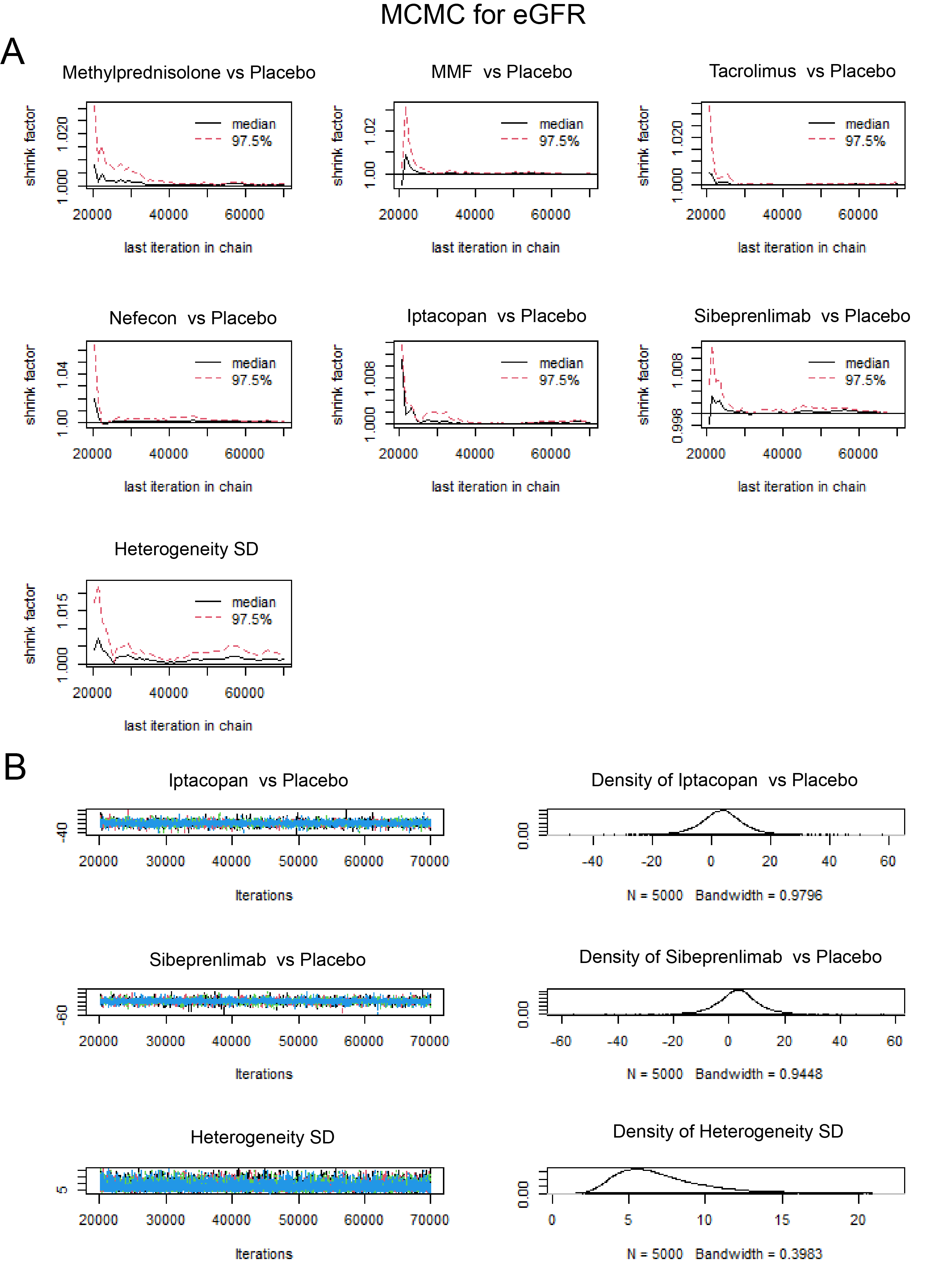


**Figure S3. Bayesian model diagnostics for the eGFR network.**

(A) Brooks–Gelman–Rubin diagnostic plot (Gelman plot) showing shrink factors close to 1.0; (B) Trace plots (left) and posterior density distributions (right) demonstrating satisfactory convergence of Markov chain Monte Carlo simulations.


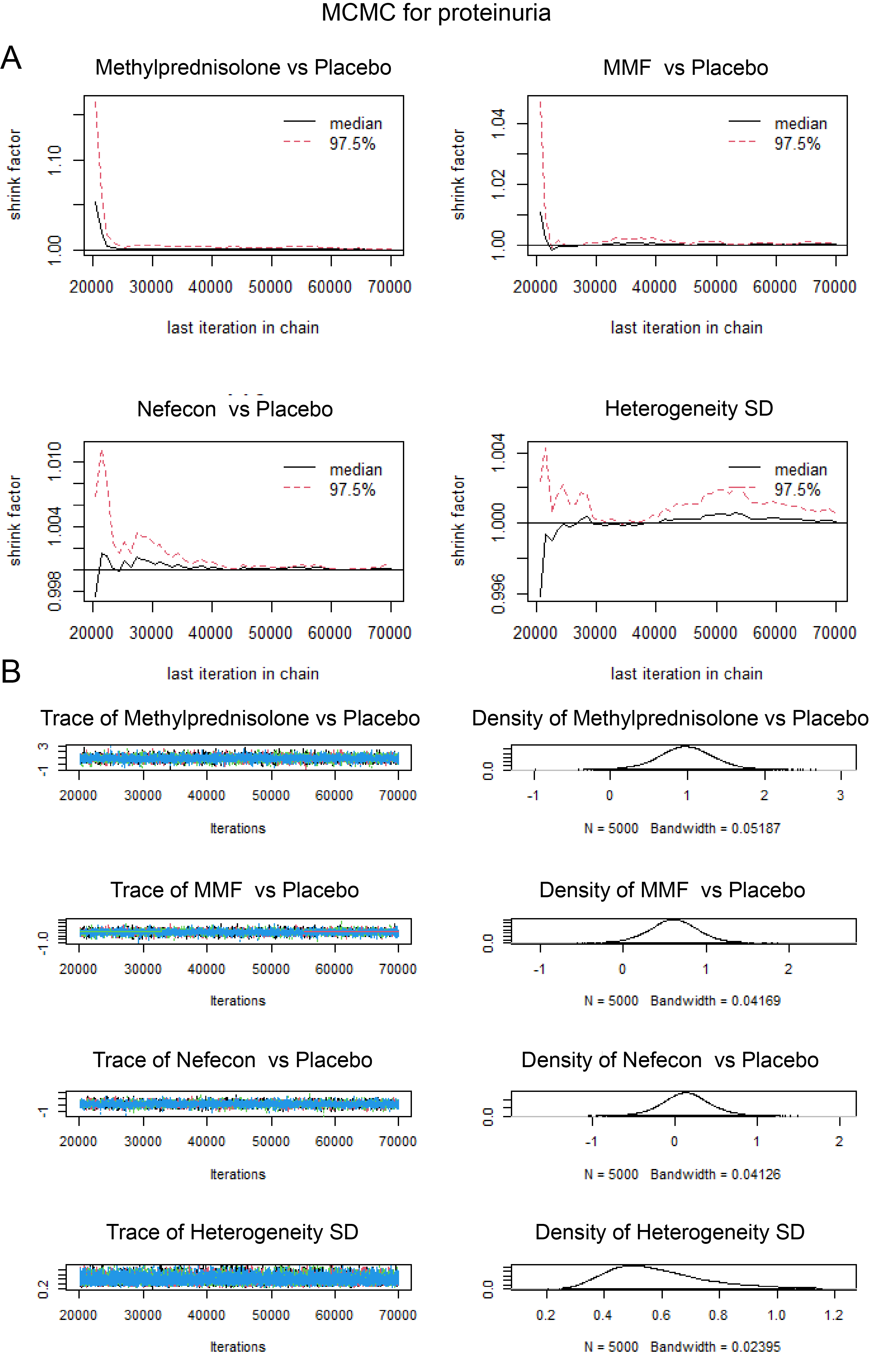


**Figure S4. Bayesian model diagnostics for the proteinuria network.**

(A) Brooks–Gelman–Rubin diagnostic plot (Gelman plot) showing shrink factors close to 1.0; (B) Trace plots (left) and posterior density distributions (right) demonstrating satisfactory convergence of Markov chain Monte Carlo simulations.


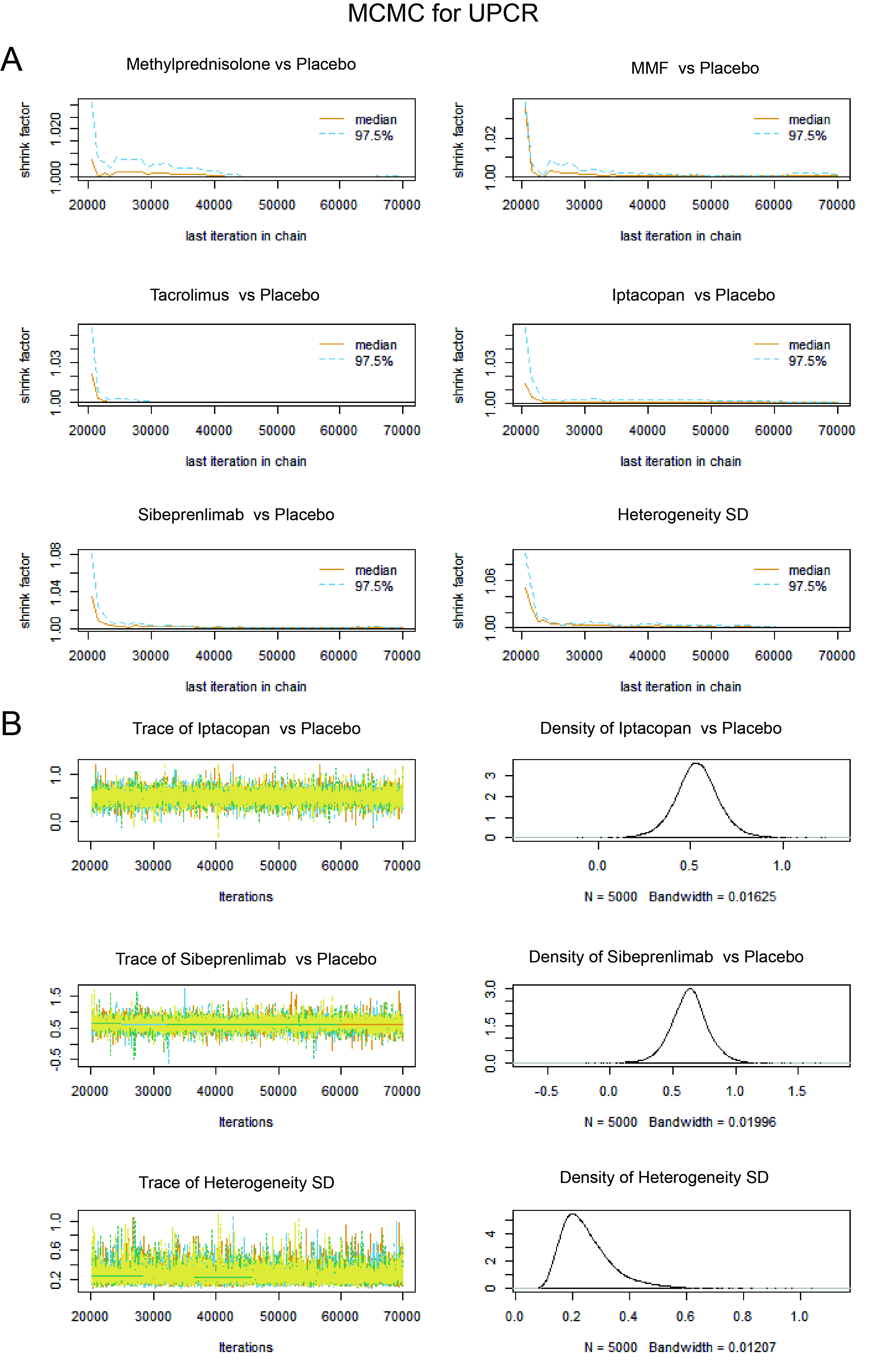


**Figure S5. Bayesian model diagnostics for UPCR network.**

(A) Brooks–Gelman–Rubin diagnostic plot (Gelman plot) showing shrink factors close to 1.0; (B) Trace plots (left) and posterior density distributions (right) demonstrating satisfactory convergence of Markov chain Monte Carlo simulations.


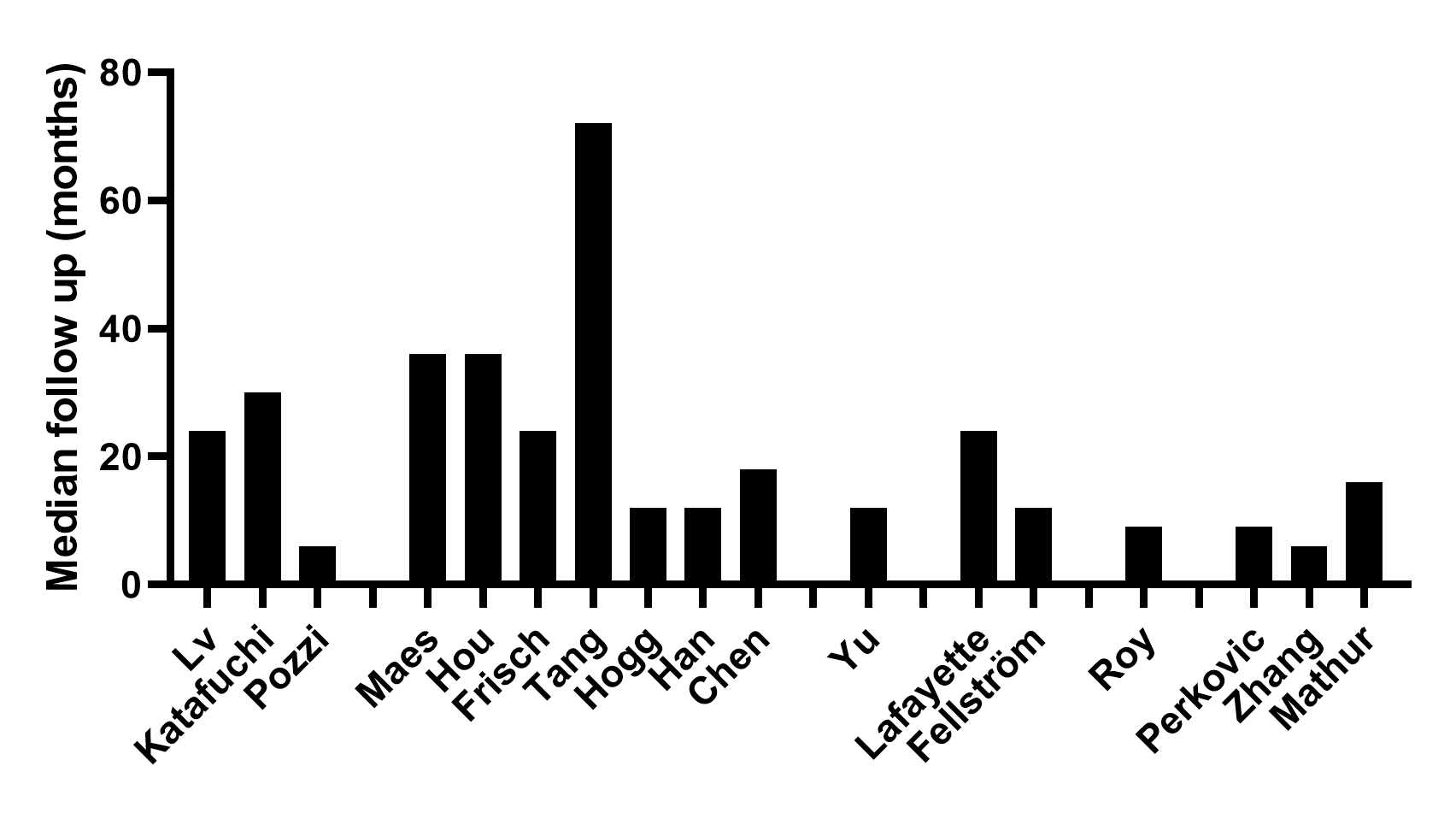


**Figure S6. Distribution of follow-up duration across included randomized controlled trials.**

Follow-up was defined as the time from randomization to the primary endpoint assessment in each trial.

**Table S1. Characteristics of included randomized controlled trials**

| Study | Setting | Drug dose (mg/day) | Median follow up (months) | Key eligibility criteria |
| --- | --- | --- | --- | --- |
| Methylprednisolone vs. Placebo |  |  |  |  |
| Lv 2022 | Japan | Oral  20 mg/d, gradually decrease to 5 mg/d | 24 | Diagnosis of IgA nephropathy confirmed by renal biopsy with sufficient tissue (minimum of 10 glomeruli) Glomerular score between 4 and 7 based on histological evaluation Age younger than 60 years Plasma creatinine concentration ≤ 1.5 mg/dL (132.6 µmol/L) No prior treatment with steroids Absence of pregnancy Exclusion: Henoch-Schönlein purpura, lupus erythematosus, diabetes mellitus, neoplasia, active peptic ulcer disease, viral hepatitis, or other infections |
| Katafuchi 2003 | America | Oral  Methylprednisolone 0.6 to 0.8mg/kg/d, followed by a tapering regimen of 8 mg/d each month | 30 | Biopsy-confirmed primary IgA nephropathy Aged 18–70 years Urinary protein excretion ≥0.75 g/24 hours with hypertension or eGFR ≥90 mL/min/1.73m² Baseline eGFR ≥30 mL/min/1.73m² Exclusion of secondary IgA nephropathy, rapidly progressive, crescentic forms or prior immunosuppressive therapy. |
| Pozzi 1999 | Italia | Intravenous and oral  Methylprednisolone 50mg/d | 6 | Histologically confirmed IgA nephropathy with mesangial IgA deposits on immunofluorescence Age between 15 and 69 years Urinary protein excretion of 1.0–3.5 g/day for at least 3 months Plasma creatinine concentration ≤ 133 μmol/L (1.5 mg/dL) No treatment with steroids or cytotoxic drugs within the previous 3 years Absence of pregnancy Exclusion: Henoch-Schönlein purpura, systemic lupus erythematosus, diabetes mellitus, neoplasia, active peptic ulcer disease, viral hepatitis, or other infections |
| MMF vs. Placebo |  |  |  |  |
| Maes 2004 | Belgium | Oral  MMF 2 g/d | 36 | Renal biopsy-proven IgA nephropathy within 5 years Age ≥18 years Presence of ≥1 risk factor: decreased renal function (insulin clearance 20-70 mL/min/1.73m²) or proteinuria >1 g/day/1.73m² or hypertension or unfavorable histology Exclusion of rapidly progressive IgAN requiring other immunosuppressants or secondary glomerulopathies or systemic diseases No immunosuppressive therapy within 6 months, pregnancy/lactation without contraception, active infections or malignancy or leukopenia or thrombocytopenia |
| Hou 2023 | China | Oral  MMF 1.5 g/d, tapered to a maintenance daily dose of 0.75-1 g/d | 36 | Biopsy-proven primary IgA nephropathy Aged 18-70 years Urinary protein excretion >1.0 g/24h eGFR <60 mL/min/1.73m² or persistent hypertension Exclusion of eGFR <30 mL/min/1.73m², secondary/familial/crescentic IgA nephropathy No prior immunosuppressive therapy Persistent proteinuria (0.75-3.5 g/24h) after 3-month run-in |
| Frisch 2005 | America | Oral  MMF 1 g/d | 24 | Biopsy-proven IgA nephropathy with samples examined by two pathologists Age 18-75 years Proteinuria ≥1 g per day while on ACEI or ARB therapy At least two of the following risk factors: male sex; hypertension ≥150/90 mmHg or requiring antihypertensive medications; creatinine clearance ≤80 ml/min at enrolment; presence of glomerulosclerosis, tubulointerstitial fibrosis or crescent formation in ≥25% of biopsy sample Creatinine clearance >20 ml/min at enrolment No malignancy, infection, liver disease, SLE, Henoch-Schoenlein purpura or other serious systemic disease or other renal biopsy diagnosis |
| Tang 2010 | China | Oral  MMF 1.5-2 g/d | 72 | Histologically confirmed IgA nephropathy with mesangial IgA deposition Persistent proteinuria over 1 g/24h on ≥3 consecutive measurements 4-6 weeks apart Age ≥18 years Serum creatinine ≤300 μmol/l (≤3.4 mg/dl) Excluded other glomerulopathies, systemic infections, or malignancy, pregnancy, lactation, or unwillingness to use contraception Excluded serum creatinine >300 μmol/l |
| Hogg 2015 | America | Oral  MMF 25-36 mg/kg/d | 12 | Biopsy-proven IgA nephropathy with mesangial immunofluorescence staining for IgA ≥ IgG and IgM Age >7 to <70 years Urine protein-creatinine ratio ≥0.6 g/g for males or ≥0.8 g/g for females Estimated glomerular filtration rate ≥50 mL/min/1.73 m² (or ≥40 mL/min/1.73 m² if on ACE inhibitor/ARB) Urine protein-creatinine ratio ≥0.6 g/g for males or ≥0.8 g/g for females post-run-in No systemic lupus erythematosus or Henoch-Schönlein purpura, chronic liver disease or significant gastrointestinal disorders, prior mycophenolate mofetil or azathioprine treatment or pregnant or breastfeeding and willing to use contraception |
| Han 2022 | Korea | Oral  MMF 1.5-2 g/d | 12 | Biopsy-proven IgA nephropathy Age 19 to 65 years Urine protein-to-creatinine ratio greater than 0.75 g per day Estimated glomerular filtration rate between 20 and 50 mL/min/1.73 m² using the Modification of Diet in Renal Disease equation Received renin-angiotensin-system blockers for at least 3 months during run-in phase No systemic inflammation or malignancy within 5 years prior to screening White blood cell count ≥3,000/mm³ No immunosuppressive therapy within 12 weeks prior to screening |
| Chen 2002 | China | Oral  MMF 1-1.5 g/d | 18 | Biopsy-confirmed IgA nephropathy Lee grade IV or V with interstitial inflammatory cell infiltration area greater than 25 percent Urinary protein excretion greater than or equal to 2 grams per 24 hours Serum creatinine less than 355 micromol per liter Exclusion of rapidly progressive glomerulonephritis syndrome or acute renal insufficiency, secondary IgA nephropathy such as Henoch-Schönlein purpura nephritis or HBV-associated nephritis or patients with significant liver impairment defined as transaminase elevation 1.5 times above normal |
| Tacrolimus vs. Placebo |  |  |  |  |
| Yu 2017 | Korea | Oral  Tacrolimus 0.1mg/kg/d, decreased to 0.05 mg/kg/d at week 8 | 12 | Biopsy-proven IgA nephropathy Age between 18 and 70 years Serum creatinine ≤1.5 mg/dL or estimated glomerular filtration rate ≥45 mL/min/1.73 m² Urine albumin to creatinine ratio ≥0.3 and <3.0 g/g creatinine Blood pressure <130/80 mmHg during the 3-month period prior to randomization |
| Nefecon vs. Placebo |  |  |  |  |
| Lafayette 2023 | Multinational | Oral  Nefecon 16mg/d | 24 | Adult patients aged 18 years or older Biopsy-confirmed primary IgA nephropathy eGFR 35–90 mL/min per 1.73 m² using CKD-EPI formula Persistent proteinuria (UPCR ≥0.8 g/g or proteinuria ≥1 g/24 hours) despite optimized supportive care Exclusion of poorly controlled diabetes (HbA1c >8%), secondary IgA nephropathy or non-IgA glomerulonephritis, poorly controlled hypertension (≥140/90 mm Hg) or kidney transplant recipients |
| Fellström 2017 | Multinational | Oral  Nefecon 8mg/d | 12 | Adult patients aged at least 18 years Biopsy-confirmed primary IgA nephropathy Urine protein creatinine ratio greater than 0.5 g/g or urinary total protein at least 0.75 g per day Estimated glomerular filtration rate at least 45 mL/min per 1.73 m² Exclusion of immunosuppressive or corticosteroid treatment within 12 months prior to randomization Exclusion of poorly controlled diabetes, severe hepatic impairment, non-IgA nephropathy glomerulonephritis or pregnant or breastfeeding women |
|  |  | Nefecon 16mg/d |  |  |
| Chaudhary 2022 | India | Oral  Nefecon 18mg/d | 9 | Biopsy-proven IgA nephropathy Persistent proteinuria >1 g/24-hour Age >18 years No immunosuppressive therapy in previous 6 months Completed 6-month run-in phase with optimized supportive care Stable renin-angiotensin system inhibition therapy Target blood pressure <130/80 mmHg during run-in Published as conference abstract only; results should be interpreted as preliminary. |
| Iptacopan vs. Placebo |  |  |  |  |
| Perkovic 2025 | Multinational | Oral (twice a day)  Iptacopan 200mg/d | 9 | Biopsy-confirmed primary IgA nephropathy Proteinuria ≥1 g/g via 24-hour urine UPCR Stable maximally tolerated RASI therapy for ≥3 months Baseline eGFR ≥30 mL/min/1.73 m² Adult patients Allowed background SGLT2 inhibitor therapy Exclusion of secondary glomerular diseases |
| Zhang 2024 | Multinational | Oral  Iptacopan 10mg/d | 6 | Biopsy-confirmed primary IgA nephropathy within 3 years eGFR ≥30 ml/min/1.73 m² Persistent proteinuria ≥0.75 g/24-hour Stable maximally tolerated RAS inhibitors ≥3 months Blood pressure controlled per KDIGO guidelines Required vaccination against encapsulated bacteria Excluded secondary glomerular diseases |
|  |  | Iptacopan 50mg/d | 6 |  |
|  |  | Iptacopan 100mg/d | 6 |  |
|  |  | Iptacopan 200mg/d | 6 |  |
| Sibeprenlimab vs. Placebo |  |  |  |  |
| Mathur 2024 | Multinational | Intravenous injection (once a month)  Sibeprenlimab 2mg/kg/d | 16 | Age 18-70 years Biopsy-proven primary IgA nephropathy Proteinuria ≥1 g/24-hour eGFR 30-90 ml/min/1.73 m² Stable renin-angiotensin system blockade No prior immunosuppressive therapy Excluded secondary glomerular diseases, diabetes mellitus or uncontrolled hypertension |
|  |  | Sibeprenlimab 4mg/kg/d | 16 |  |
|  |  | Sibeprenlimab 8mg/kg/d | 16 |  |

Footnote: Characteristics of randomized controlled trials included in the network meta-analysis, including study setting, drug regimen, follow-up duration, and key eligibility criteria.

**Table S2. Baseline characteristics of patients across trials**

| Study | Number of patients | | | Age | | Male | | BMI | | eGFR (ml/min/1.73 m2) | | proteinuria(g/d) OR UPCR (g/g)* | |
| --- | --- | --- | --- | --- | --- | --- | --- | --- | --- | --- | --- | --- | --- |
|  | Total | I | C | I | C | I | C | I | C | I | C | I | C |
| Methylprednisolone vs. Placebo |  |  |  |  |  |  |  |  |  |  |  |  |  |
| Lv 2022 | 90 | 43 | 47 | 33.6±13.4 | 32.5±10.8 | 15 | 22 | 24.4±4.5 | 23.4±3.7 | NA | | 2.52±2.21 | 1.43±0.92 |
| Katafuchi 2003 | 503 | 257 | 246 | 35.6±12.5 | 36.6±12.5 | 155 | 150 | NA | | 56.1±23.6 | 59.0±26.4 | 1.99±1.28 | 1.93±1.11 |
| Pozzi 1999 | 86 | 43 | 43 | 36.8±4.8 | 40±5.5 | 30 | 31 | NA | | NA | | 1.85±0.25 | 2±0.2 |
| MMF vs. Placebo |  | | |  |  |  |  |  |  |  |  |  |  |
| Maes 2004 | 34 | 21 | 13 | 39±11 | 43±15 | 16 | 8 | NA | | 73±5 | 69±7 | 1.9±0.3 | 1.3±0.4 |
| Hou 2023 | 170 | 85 | 85 | 35.0±8.7 | 38.2±9.8 | 42 | 52 | 23.3±3.1 | 22.9±3.7 | 50.9±18.2 | 49.3±17.7 | 2.1±1.9 | 1.7±1.3 |
| Frisch 2005 | 32 | 17 | 15 | 42.3±13.3 | 38.8±9.3 | 16 | 11 | 31±12.4 | 28±6.4 | 38.8±22.2 | 41±26.3 | 2.7±1.6 | 2.7±1.4 |
| Tang 2010 | 40 | 20 | 20 | 42.1±2.6 | 43.3±2.8 | 6 | 8 | NA | | 52.5±4.4 | 50±4.5 | 1.8±0.21 | 1.87±0.28 |
| Hogg 2015 | 52 | 25 | 27 | 31.8±11.7 | 32.2±13.2 | 14 | 18 | NA | | 95.3±36.5 | 105.6±49.0 | 1.59±0.9* | 1.40±0.56* |
| Han 2022 | 44 | 24 | 20 | 44.0±10.6 | 46.1±7.8 | 15 | 10 | 24.5±3.9 | 24.3±3.5 | 36.3±9.4 | 33.0±7.7 | 1.7±0.6* | 2.2±1.0* |
| Chen 2002 | 62 | 31 | 31 | 28±10 | 29±10 | 25 | 22 | NA | | NA | | 3.2±1.7 | 2.9±1.5 |
| Tacrolimus vs. Placebo |  |  |  |  |  |  |  |  |  |  |  |  |  |
| Yu 2017 | 37 | 18 | 19 | 36.8±11.3 | 41.0±12.6 | 6 | 5 | NA | | 77.4±22.9 | 83.1±24.1 | 1.2±0.36* | 1.45±0.53* |
| Nefecon vs. Placebo |  |  |  |  |  |  |  |  |  |  |  |  |  |
| Lafayette 2023 | 364 | 182 | 182 | 43.0±3.5 | 41.8±3.8 | 117 | 123 | 28.0±5.2 | 27.0±4.4 | 56.1±18.9 | 55.1±16.1 | 1.77±0.93 | 1.70±1.05 |
| Fellström 2017 | 101 | 51 | 50 | 40.6±13.0 | 38.9±12.0 | 37 | 35 | 26.5±4.4 | 27.5±5.4 | 65.1±28.8 | 61.5±21.2 | 1.00±0.67 | 1.10±1.04 |
|  | 98 | 48 | 50 | 37.5±11.9 | 38.9±12.0 | 33 | 35 | 27.8±5.2 | 27.5±5.4 | 56.1±18.9 | 55.1±16.1 | 1.10±0.74 | 1.10±1.04 |
| Chaudhary 2022 | 53 | 27 | 26 | 32.8±11.4 | 31.0±9.9 | 15 | 15 | 21.7±3.1 | 21.5±3.2 | 80.0±19.8 | 82.2±15.6 | 2.44±1.05 | 2.39±1.06 |
| Iptacopan vs. Placebo |  |  |  |  |  |  |  |  |  |  |  |  |  |
| Perkovic 2025 | 250 | 125 | 125 | 39.3±12.4 | 39.6±12.6 | 71 | 60 | NA | | 62.7±26.0 | 65.5±26.7 | 1.80±0.96* | 1.90±0.96* |
| Zhang 2024 | 45 | 20 | 25 | 39.2±12.4 | 39.4±11.0 | 9 | 18 | 26.5±4.4 | 27.5±5.4 | 66.0±28.5 | 65.7±32.6 | 1.90±1.10* | 1.30±0.60* |
|  | 44 | 19 | 25 | 36.6±8.4 | 39.4±11.0 | 13 | 18 | 27.8±5.2 | 27.5±5.4 | 53.8±22.7 | 65.7±32.6 | 1.70±0.80* | 1.30±0.60* |
|  | 47 | 22 | 25 | 36.0±13.2 | 39.4±11.0 | 11 | 18 | 29±1.5 | 27.8±1.8 | 67.0±31.8 | 65.7±32.6 | 1.80±0.90* | 1.30±0.60* |
|  | 51 | 26 | 25 | 42.5±15.8 | 39.4±11.0 | 15 | 18 | 21.7±3.1 | 21.5±3.2 | 57.9±28.9 | 65.7±32.6 | 1.30±1.00* | 1.30±0.60* |
| Sibeprenlimab vs. Placebo |  |  |  |  |  |  |  |  |  |  |  |  |  |
| Mathur 2024 | 76 | 38 | 38 | 44.5±11.5 | 35.5±8.5 | 22 | 14 | 27.2±4.5 | 27.4±6.7 | 76.3±29.8 | 71.5±20.8 | 1.46±0.12* | 1.68±0.17* |
|  | 79 | 41 | 38 | 42.8±13.3 | 35.5±8.5 | 26 | 14 | 28.1±6.4 | 27.4±6.7 | 74.0±24.5 | 71.5±20.8 | 1.53±0.12* | 1.68±0.17* |
|  | 76 | 38 | 38 | 44.8±12.3 | 35.5±8.5 | 26 | 14 | 27.6±5.8 | 27.4±6.7 | 58.3±18.8 | 71.5±20.8 | 1.44±0.14* | 1.68±0.17* |

Footnote:I, Intervention group; C, Control (supportive care) group; BMI, body mass index; eGFR, estimated glomerular filtration rate; UPCR, urinary protein-to-creatinine ratio; NA, not available.

**Table S3. GRADE evaluation for all comparisons between the different treatments using CINeMA**

| Comparison | Number of studies | Within-study bias | Reporting bias | Indirectne | Heterogeneit | Imprecision | Confidence rating* |
| --- | --- | --- | --- | --- | --- | --- | --- |
| SAE | | | | | | | |
| ME:PL | 6 | Some concerns | Low risk | Low risk | Low risk | High risk | Low |
| MMF:PL | 8 | Some concerns | Low risk | Low risk | Low risk | High risk | Low |
| NEF:PL | 4 | Low risk | Low risk | Low risk | Low risk | Some concerns | Moderate |
| IP:PL | 1 | Low risk | Low risk | Low risk | Low risk | High risk | Low |
| SI:PL | 1 | Low risk | Low risk | Low risk | Low risk | High risk | Low |
| eGFR | | | | | | |  |
| ME:PL | 3 | Some concerns | Low risk | Low risk | Low risk | Some concerns | Moderate |
| MMF:PL | 5 | Some concerns | Low risk | Low risk | Low risk | High risk | Low |
| TA:PL | 2 | Some concerns | Low risk | Low risk | Low risk | High risk | Low |
| NEF:PL | 5 | Low risk | Low risk | Low risk | Low risk | Some concerns | Moderate |
| IP:PL | 1 | Low risk | Low risk | Low risk | Low risk | Low risk | Moderate |
| SI:PL | 1 | Low risk | Low risk | Low risk | Low risk | Low risk | Moderate |
| Proteinuria | | | | | | |  |
| ME:PL | 3 | Some concerns | Some concerns | Low risk | Low risk | High risk | Low |
| MMF:PL | 6 | Some concerns | Some concerns | Low risk | Low risk | Some concerns | Low |
| NEF:PL | 4 | Low risk | Some concerns | Low risk | Low risk | Low risk | Moderate |
| UPCR | | | | | | |  |
| ME:PL | 1 | Some concerns | High risk | Low risk | High risk | Some concerns | Very Low |
| MMF:PL | 2 | Some concerns | Some concerns | Low risk | Low risk | Low risk | Moderate |
| TA:PL | 2 | Some concerns | Some concerns | Low risk | Low risk | Low risk | Moderate |
| NEF:PL | 1 | Low risk | High risk | Low risk | High risk | Low risk | Very Low |
| IP:PL | 2 | Low risk | Some concerns | Low risk | Low risk | Low risk | Moderate |
| SI:PL | 1 | Low risk | Some concerns | Low risk | Low risk | Low risk | Moderate |

Footnote: Our judgements described below are based on the recommendations of the online documentation of CINeMA (https://cinema.ispm.unibe.ch). The overall confidence rating was determined according to the following criteria: all five domains with “Low risk”: high confidence; one or two domains with “some concerns”: moderate; three domains with “some concerns”: Low; one domain with “High risk”: Low, two domain with “High risk”: Very Low. PL = Placebo, ME = Methylprednisolone, TA = Tacrolimus, NEF = Nefecon, IP = Iptacopan, SI = Sibeprenlimab.

**Table S4. Subgroup analyses by baseline eGFR**

| Drug | Baseline eGFR < 60 ml/min/1.73m² (RR/MD, 95%CI) | Baseline eGFR≥ 60 ml/min/1.73m² (RR/MD, 95%CI) | P for interaction |
| --- | --- | --- | --- |
| **SAE** |  |  |  |
| MMF | 1.77 (0.45, 6.98) | 1.33 (0.75, 2.35) | 0.62 |
| Nefecon | 1.89 (0.78, 4.59) | NA | NA |
| Iptacopan | 0.73 (0.15, 3.53) | NA | NA |
| Sibeprenlimab | NA | 1.00 (0.06, 15.41) | NA |
| **eGFR slope** |  |  |  |
| MMF | 2.82 (-1.30, 6.94) | -3.70 (-8.30, 1.10) | 0.03 |
| Nefecon | 2.40 (2.20, 2.60) | 10.71 (-4.72, 26.14) | 0.20 |
| Iptacopan | 3.5 (1.3-5.7) | NA | NA |
| Sibeprenlimab | NA | 3.30 (2.70, 3.80) | NA |
| **proteinuria** |  |  |  |
|  |  |  |  |
| MMF | -0.86 (-1.12, -0.60) | NA | NA |
| Nefecon | -0.24 (-0.31, -0.17) | NA | NA |
| **UPCR** |  |  |  |
| Iptacopan | -0.50 (-0.70, 0.30) | NA | NA |
| Sibeprenlimab | NA | -0.55 (-0.84, -0.26) | NA |

Footnote: Subgroup analyses by baseline eGFR (<60 vs.≥60 ml/min/1.73m²). Results are shown as risk ratios or mean differences with 95% confidence intervals. NA indicates that an estimate could not be generated for that specific treatment-subgroup combination, primarily due to insufficient data points within the network meta-analysis model to produce a reliable estimate.

**Table S5. Strengths, limitations, and safety profiles of included trials**

| Study | Main Strengths | Main Limitations | Safety Profile Summary |
| --- | --- | --- | --- |
| Methylprednisolone vs. Placebo |  |  |  |
| Lv 2022 | Prospective, randomized, controlled design. Well-defined histological inclusion criteria. Long follow-up period. Detailed proteinuria monitoring and subgroup analysis. | Baseline UP-UCR higher in steroid group. Small sample size, no power calculation. Possibly insufficient steroid dose to affect kidney survival. | Palpitations, increased sweating, insomnia and facial blushing |
| Katafuchi 2003 | Large-scale, international, multicenter, double-blind, randomized clinical trial. Event-driven design with long-term follow-up. Protocol adapted in response to early safety signals. Significant reduction in the primary composite kidney outcome. | The study protocol was modified mid-trial due to excess serious infections, leading to two different dosing regimens. This modification may introduce heterogeneity when comparing results across the full and reduced-dose cohorts. | Serious infections, new-onset diabetes, gastrointestinal hemorrhage, fracture or osteonecrosis and cardiovascular events |
| Pozzi 1999 | Prospective randomized controlled trial. Long-term follow-up up to 10 years. Use of a combined intravenous and oral steroid regimen to minimize side effects. | Relatively small sample size. Imbalance in baseline macrohaematuria between groups. | Increased body weight, new-onset hypertension and new-onset diabetes mellitus. |
| MMF vs. Placebo |  |  |  |
| Maes 2004 | Prospective, placebo-controlled, randomized study design. Use of objective renal function measure (inulin clearance) and detailed immunological monitoring. High treatment compliance as measured by MPA levels. | Small sample size. Underpowered to definitively prove or exclude a treatment effect. Excluded patients with rapidly progressive IgA nephropathy, limiting generalizability. | Reactivation of pulmonary tuberculosis, gastrointestinal complaints, transient leukopenia, pregnancy, and death from metastatic rectal carcinoma. |
| Hou 2023 | Prospective randomized open-label trial with blinded endpoint assessment. Included high-risk patient population with moderately reduced eGFR. Long-term follow-up including a post-trial observation period. | Open-label design. Excluded patients with proteinuria greater than 3.5 g per day. Single-center study conducted in a Chinese population, may limit generalizability. | Gastrointestinal symptoms such as abdominal distension and diarrhea, and infections predominantly pneumonia. |
| Frisch 2005 | Double-blind, randomized, placebo-controlled trial design. Multicenter study focusing on a North American patient population. Enrolled patients at high risk for disease progression, suitable for assessing intervention effect. | Small sample size. Trial was terminated early due to a trend towards worse outcomes in the treatment group. Study population had moderately advanced renal insufficiency at baseline, potentially limiting treatment benefit. | Gastrointestinal side effects leading to therapy discontinuation, and one deep vein thrombosis in the placebo group. |
| Tang 2010 | Long-term follow-up. Focused on a specific high-risk patient population with persistent proteinuria despite maximal angiotensin blockade but mild histological lesions. Use of a composite renal endpoint. | Small sample size. Potential baseline imbalance with slightly higher proteinuria and lower eGFR in the control group. Results may primarily apply to a Chinese patient population. | Decreased hemoglobin, diarrhea, transient upper gastrointestinal upset, and infections such as urinary tract infection and cervical lymphadenitis. |
| Hogg 2015 | Multicenter, double-blind, placebo-controlled RCT design. Stepwise approach using non-immunosuppressive therapy first. Utilized Oxford classification for standardized biopsy assessment. Included a range of ages from children to adults. | Low patient enrollment. Short follow-up period. | Malignant melanoma, multiple abdominal injuries, nausea. |
| Han 2022 | Focused on a specific high-risk population with advanced IgAN. Investigated combination therapy of MMF with corticosteroids. Multicenter, randomized study design. | Small sample size due to recruitment difficulties. High dropout rate. Imbalanced baseline proteinuria between groups. | Sudden death of unknown cause, urinary tract infection, foot fracture, and shoulder and cervical sprain. |
| Chen 2002 | Focused on a specific high-risk population with severe IgAN. Long-term follow-up. Included repeat renal biopsy data showing histopathological improvement. Evaluated effects on serum lipids. | Small sample size. Open-label design. High cost of MMF therapy mentioned as a practical constraint. | Diarrhea, herpes zoster, nausea, and transient elevated liver enzymes. |
| Tacrolimus vs. Placebo |  |  |  |
| Yu 2017 | Long-term observational follow-up of 5 years after drug cessation. Follows a prior randomized controlled trial design. Examines the persistence of effect after discontinuation of therapy. | Small sample size. Population restricted to IgAN patients with mild to moderate proteinuria. Lacks post-treatment biopsy data for histopathological correlation. | General weakness and myalgia. |
| Nefecon vs. Placebo |  |  |  |
| Lafayette 2023 | Large, multicenter, randomized, double-blind, placebo-controlled phase 3 trial. Long 2-year follow-up including a 15-month observation period after treatment. Evaluated a novel, disease-specific therapy targeting the gut-kidney axis. Demonstrated consistent benefit across patient subgroups. | Used surrogate endpoints rather than long-term clinical kidney failure outcomes. Lack of data for patients with proteinuria below the inclusion threshold. Study population was predominantly White, with no Black or African American patients enrolled. | One death due to SARS-CoV-2 infection, cerebral hemorrhage, Community-acquired pneumonia and Campylobacter colitis, hypertension, and peripheral edema. |
| Fellström 2017 | First randomized double-blind placebo-controlled trial demonstrating TRF-budesonide reduces proteinuria and stabilizes eGFR in IgA nephropathy. Includes pre-planned interim analysis and sustained follow-up showing lasting treatment effect. All patients maintained optimized RAS blockade throughout. | Small sample size. Study population predominantly white. Short treatment duration. No histopathology data available to assess correlation with renal outcomes. | Deep vein thrombosis, unexplained worsening of renal function, increased proteinuria, and decline in renal function. |
| Chaudhary 2022 | First open-label RCT evaluating TRF-budesonide specifically in an Indian IgA nephropathy population. Includes a 6-month follow-up phase after treatment cessation to assess sustained effect. | Small sample size. Lack of detailed baseline pathological characteristics. No analysis of histological outcomes. | Serious adverse events were not specifically detailed. |
| Iptacopan vs. Placebo |  |  |  |
| Perkovic 2025 | Large Phase 3 multicenter randomized double-blind placebo-controlled trial. Pre-specified interim analysis with robust statistical design. Includes subgroup analyses across demographics, geography, baseline proteinuria, eGFR, and SGLT2i use. Uses 24-hour urine collection for accurate proteinuria measurement. | Interim analysis at 9 months only, full 24-month outcomes not yet available. Study population predominantly Asian. Interim analysis includes only first 250 patients reaching Month 9 visit. Does not report histological outcomes or correlation with clinical response. | specific types of SAEs were not detailed in this analysis. |
| Zhang 2024 | Phase 2 adaptive design allowing dose/sample size adjustments based on interim analysis. Multicenter randomized double-blind placebo-controlled trial across diverse ethnicities. Comprehensive biomarker analysis confirming target engagement and mechanism of action. Includes both 3-month and 6-month treatment data for dose-response evaluation. | Excluded patients with eGFR below 30 mL/min per 1.73 m². Relatively short treatment duration. Not powered to test efficacy of individual doses, only dose-response relationship. Small sample size. | Serious adverse events included COVID-19 infection, and inhalation of chlorine gas. Both events were considered unrelated to study treatment. |
| Sibeprenlimab vs. Placebo |  |  |  |
| Mathur 2024 | Sub-analysis focusing on Chinese population within a global trial. Includes 2-year follow-up data for eGFR and proteinuria. Double-blind, randomized, placebo-controlled design. Patients on stable optimized supportive therapy. | Small sample size. Post-hoc analysis of a larger trial population. No detailed histological outcome data. Limited generalizability beyond Chinese ethnicity. | specific types of SAEs were not detailed in this analysis. |

Footnote: This table provides a qualitative, study-level synthesis of key methodological attributes and safety observations. It is intended to contextualize the evidence within the network meta-analysis.
